# RB1 loss triggers dependence on ESRRG in retinoblastoma

**DOI:** 10.1126/sciadv.abm8466

**Published:** 2022-08-19

**Authors:** Matthew G. Field, Jeffim N. Kuznetsoff, Michelle G. Zhang, James J. Dollar, Michael A. Durante, Yoseph Sayegh, Christina L. Decatur, Stefan Kurtenbach, Daniel Pelaez, J. William Harbour

**Affiliations:** ^1^ Bascom Palmer Eye Institute, Sylvester Comprehensive Cancer Center, and Interdisciplinary Stem Cell Institute, University of Miami Miller School of Medicine, Miami, FL 33136, USA.; ^2^ Department of Ophthalmology and Visual Sciences, University of Iowa, Iowa City, IA 52242, USA.; ^3^ Department of Ophthalmology and Simmons Comprehensive Cancer Center, University of Texas Southwestern Medical Center, Dallas, TX 75390, USA.

## Abstract

Retinoblastoma (Rb) is a deadly childhood eye cancer that is classically initiated by inactivation of the RB1 tumor suppressor. Clinical management continues to rely on nonspecific chemotherapeutic agents that are associated with treatment resistance and toxicity. Here, we analyzed 103 whole exomes, 20 whole transcriptomes, 5 single-cell transcriptomes, and 4 whole genomes from primary Rb tumors to identify previously unknown Rb dependencies. Several recurrent genomic aberrations implicate estrogen-related receptor gamma (ESRRG) in Rb pathogenesis. RB1 directly interacts with and inhibits ESRRG, and RB1 loss uncouples ESRRG from negative regulation. ESRRG regulates genes involved in retinogenesis and oxygen metabolism in Rb cells. ESRRG is preferentially expressed in hypoxic Rb cells in vivo. Depletion or inhibition of ESRRG causes marked Rb cell death, which is exacerbated in hypoxia. These findings reveal a previously unidentified dependency of Rb cells on ESRRG, and they implicate ESRRG as a potential therapeutic vulnerability in Rb.

## Teaser

ESRRG as an essential mediator of hypoxic adaptation and cell survival in retinoblastoma offers a promising therapeutic target.

## INTRODUCTION

Retinoblastoma (Rb) is the most common pediatric eye cancer and an important cause of childhood cancer death worldwide (*1*). Despite considerable improvements in treatment and patient survival over the past century (*2*), severe visual impairment and loss of the eye are still common, due in part to treatment resistance and toxicity associated with currently used chemotherapeutic agents. A better understanding of molecular dependencies in Rb could lead to more specific and effective targeted therapies. Rb is almost always initiated by mutational inactivation of the RB1 tumor suppressor in a susceptible retinal progenitor cell (*3*, *4*), and it is generally thought that subsequent aberrations are required for full malignant transformation (*5*, *6*). Several recurrent genomic events have been described in Rb, including mutations in *BCOR* and *CREBBP*, and chromosome copy number variations (CNVs) affecting 1q, 2p, 6p, 13q, and 16q (*7*–*12*). However, there has been limited functional validation and no clinically relevant targeted therapies associated with any proposed secondary drivers. Here, we searched for functional drivers of Rb progression that could represent potential therapeutic targets by performing a large integrative multi-omics analysis of data from whole-exome sequencing (WES), whole-genome sequencing (WGS), RNA sequencing (RNA-seq), single-cell RNA-seq (scRNA-seq), and other methods. We found that the estrogen-related receptor (ESRR) gamma (ESRRG) is an essential mediator of hypoxic adaptation and cell survival in Rb that is constitutively activated by RB1 loss and is subsequently affected by recurrent genomic aberrations in Rb. These findings suggest a selective pressure to increase ESRRG activity during Rb progression and offer a potential target for therapy.

## RESULTS

### Genomic landscape of Rb implicates ESRRG in tumor progression

**Fig. 1.**
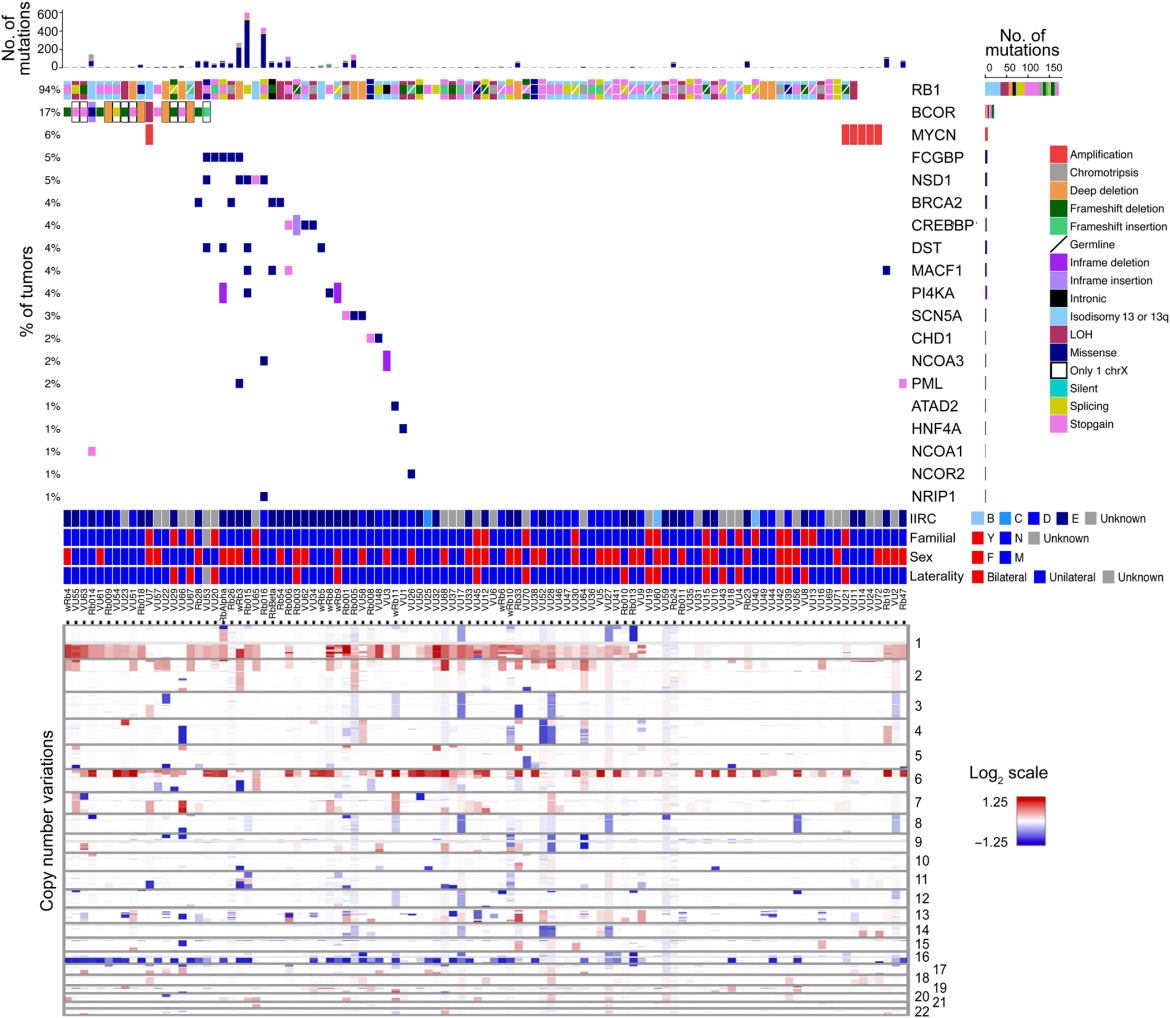
Genomic landscape of 103 primary Rbs. Oncoprint of 103 primary Rb samples analyzed by WES. Data include status of the most commonly mutated genes, types of mutations, International Intraocular Retinoblastoma Classification (IIRC) status, family history of Rb, gender, laterality, and chromosome copy number aberrations. LOH, loss of heterozygosity.

**Fig. 2.**
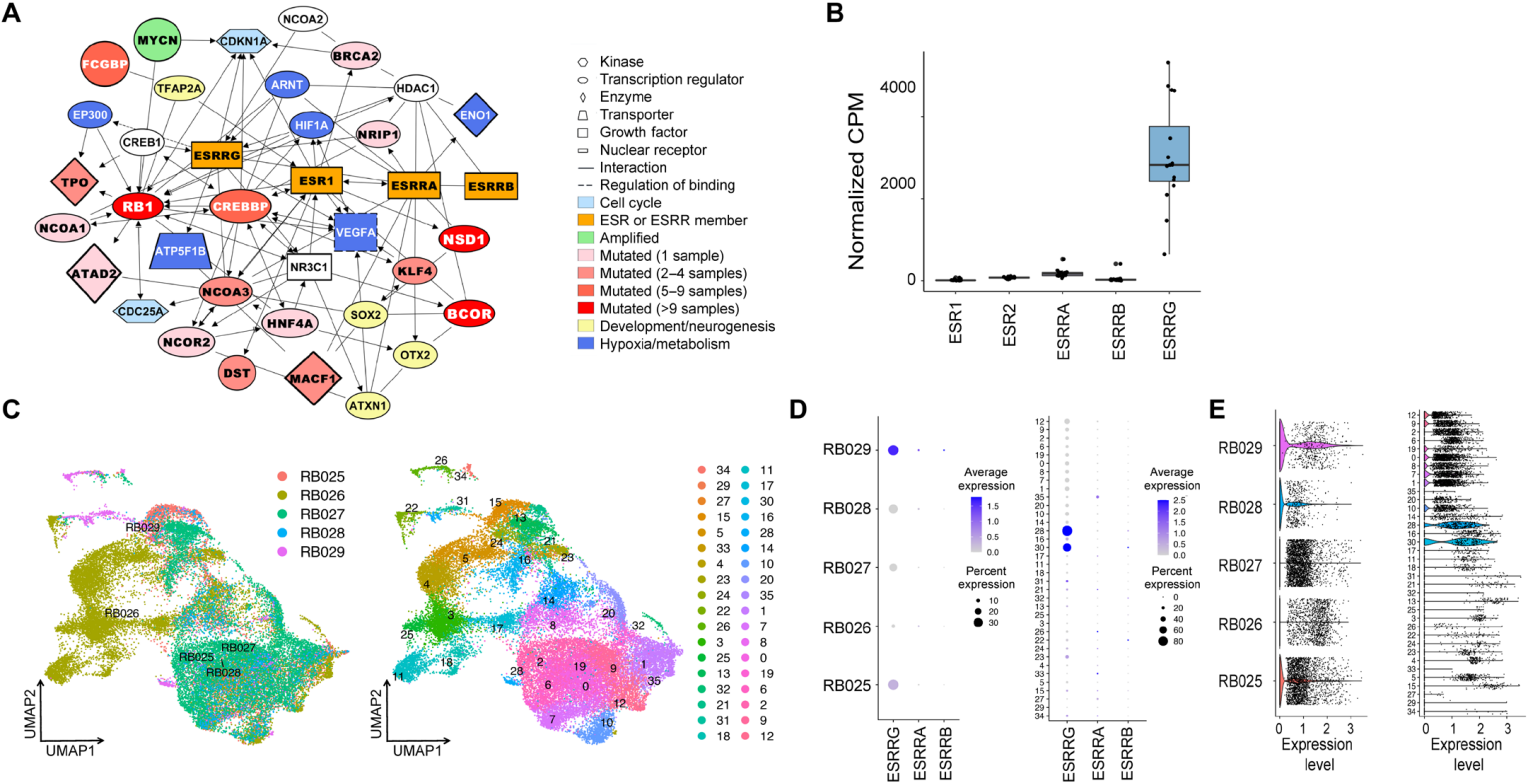
Recurrent genomic aberrations point to ESRRG as a common target for deregulation in Rb. (**A**) Ingenuity Pathway Analysis of the most common mutations in 103 Rb samples. (**B**) RNA-seq analysis of mRNA expression for ESR/ESRR family members in 16 primary Rb tumors. (**C**) Uniform manifold approximation and projection (UMAP) plots of scRNA-seq data from five primary Rb tumor samples obtained at enucleation, sorted by tumor sample (left) and Seurat cluster (right). (**D**) Dot plot analysis of scRNA-seq data showing relative single-cell expression of *ESRRA*, *ESRRB*, and *ESRRG* by tumor sample (left) and Seurat cluster (right). (**E**) Violin plots of scRNA-seq data showing *ESRRG* expression by tumor sample (left) and Seurat cluster (right). CPM, counts per million.

WES data from 103 primary Rb samples were analyzed (Fig. 1, fig. S1, and data S1). Mutations were detected in *RB1* in 94% of samples, including a stop-gain in 41 cases (39.8%), isodisomy of chromosome 13q in 36 cases (35.0%), loss of heterozygosity of *RB1* in 19 cases (18.5%), splicing mutations in 20 cases (19.4%), homozygous deletion of *RB1* in 9 cases (8.8%), and chromothripsis involving the *RB1* locus in 9 cases (8.8%). Other recurrent mutations were found in *BCOR* (17%); *FCGBP* (5%); *NSD1* (5%); *BRCA2*, *CREBBP*, *DST*, *MACF1*, and *PI4KA* (4% for each); *SCN5A* (3%); and *CHD1*, *NCOA3*, and *PML* (2% for each). The most common CNVs included 1q gain (67%), 6p gain (66%), 16q loss (56%), 2p gain (44%), 16p loss (29%), and 7q gain (26%). The *MYCN* locus at 2p24 was amplified in six cases, three of which had no detectable *RB1* mutation. We next searched for functional patterns in the genomic landscape using Ingenuity Pathway Analysis (*13*). Strikingly, most of the proteins encoded by recurrently mutated genes participate in an estrogen receptor/ESRR (ESR/ESRR) protein interaction network involved in development, neurogenesis, metabolism, hypoxia, and cell cycle regulation (Fig. 2A). *ESRRG* was the only ESR/ESRR family member that was highly expressed by RNA-seq in 16 primary Rb tumor samples (Fig. 2B). By scRNA-seq, *ESRRG* was the predominant ESR/ESRR family member expressed in primary Rb tumor cells (Fig. 2, C to E, and fig. S2). *ESRRG* is located on chromosomal region 1q41, which was the most common target of arm-level gain (Fig. 1). In addition, two of four Rb samples available for analysis by WGS and matched RNA-seq revealed more complex chromosomal rearrangements near the *ESRRG* locus associated with increased *ESRRG* mRNA expression compared to the two samples without 1q alterations (fig. S3). These findings suggest that mutations and genomic aberrations that affect *ESRRG* are common in Rb.

### ESRRG regulates genes involved in retinogenesis and oxygen metabolism in Rb cells

To gain further insight into how ESRRG regulation may drive Rb progression, we performed chromatin immunoprecipitation (ChIP) using an anti-ESRRG antibody followed by DNA sequencing [ChIP sequencing (ChIP-seq)] in our RB006 and RB018 low-passage Rb cell lines (fig. S4A). A high-confidence dataset of 8163 significantly enriched peaks shared between both RB006 and RB018 was used for downstream analyses (fig. S4B and data S2). ESRRG was enriched not only at ERRE transcriptional regulatory motifs, as expected, but also at binding motifs associated with retinal and neuronal transcription factors CRX, OTX2, NEUROD, and LHX family members (fig. S4C and data S2). Many ESRRG peaks occurred in regulatory regions containing one or more of these other binding motifs but not an ERRE motif, suggesting that ESRRG can interact with chromatin independently of its canonical binding site. Using peak location analysis, ~15% of ESRRG peaks occurred in promoter regions [±3 kb from the transcription start site (TSS)], whereas almost half occurred at presumed enhancer regions between 10 and 100 kb from the TSS, with ESRRG peaks occurring at 446 (24.3%) of 1834 annotated retinal/neural super-enhancer regions (fig. S4C) (*14*).

**Fig. 3.**
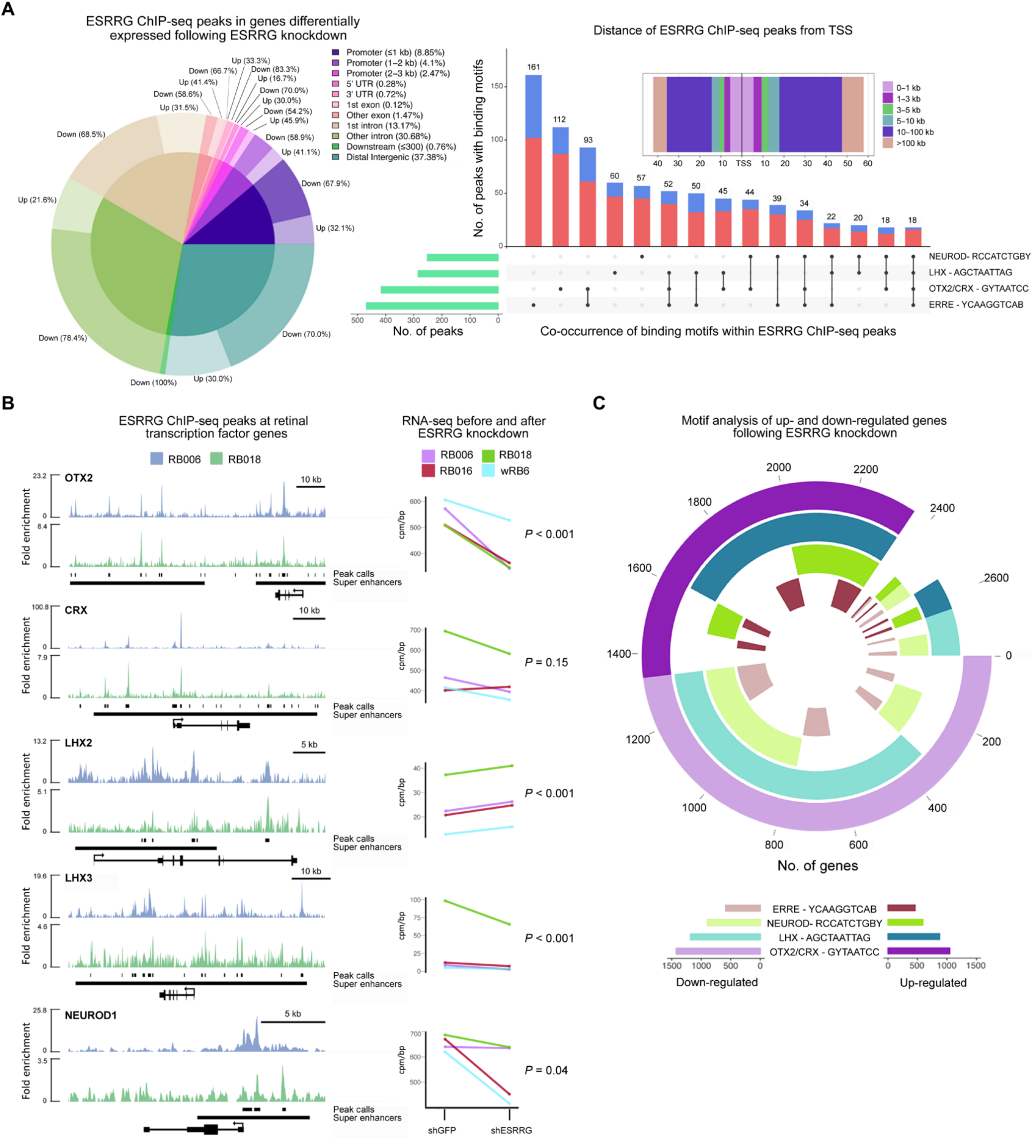
ESRRG regulates genes involved in retinogenesis and oxygen metabolism in Rb. (**A**) Integrated analysis of ESRRG ChIP-seq peaks and RNA-seq differentially expressed genes with or without shRNA-mediated knockdown of ESRRG (shESRRG). ChIP-seq was performed in RB006 and RB018 cells, and RNA-seq was performed in RB006, RB016, RB018, and wRB6 cells. The pie chart displays the percentage of peaks located within gene regions that are associated with differentially expressed genes (inner circle) or up-regulated and down-regulated genes (outer donut). The rectangular plot (top right) exhibits peak distance from the TSS. The bar plot shows the presence and co-occurrence of the most significantly enriched transcription factor binding motifs (FDR < 0.05) found within the ESRRG ChIP-seq peaks associated with up-regulated (blue) or down-regulated (red) genes after ESRRG knockdown. UTR, untranslated region. (**B**) ESRRG ChIP-seq track plots and corresponding RNA-seq data with (shESRRG) and without (shGFP) ESRRG knockdown at key retinal transcription factors involved in retinal development and differentiation. (**C**) Presence and co-occurrence of ERRE, NEUROD, LHX, and CRX/OTX2 binding motifs within ±3 kb from the TSS of all significantly up-regulated (darker colors) and down-regulated (lighter colors) genes (FDR < 0.05) following ESRRG knockdown. Individual genes are depicted as radii, and each donut represents the indicated binding motif.

To correlate ESRRG chromatin localization with gene expression, we performed RNA-seq before and after depletion of ESRRG in RB006 and RB018 cells, and in two more of our recently established Rb cell lines (RB016 and wRB6). Of the 8163 significant ESRRG ChIP-seq peaks, 1226 were associated with 738 differentially expressed genes [false discovery rate (FDR) < 0.05], including 249 up-regulated genes (353 peaks) and 489 down-regulated genes (873 peaks) (data S3). Up-regulated genes were enriched for pathways related to cell cycle regulation and oxidative phosphorylation, whereas down-regulated genes were enriched for pathways related to neurogenesis, hypoxia, and estrogen signaling. CRX, OTX2, NEUROD, and LHX binding motifs were enriched in both up- and down-regulated genes, with 18.0% of peaks located in promoter regions (±3 kb from the TSS) and 52% located 10 to 100 kb from the TSS (Fig. 3, A to D, and fig. S5). Consistent with these findings, scRNA-seq analysis demonstrated a significant correlation between Rb cells expressing *ESRRG* and those expressing *CRX* and *OTX2* (fig. S6).

### RB1 interacts with and inhibits ESRRG

**Fig. 4.**
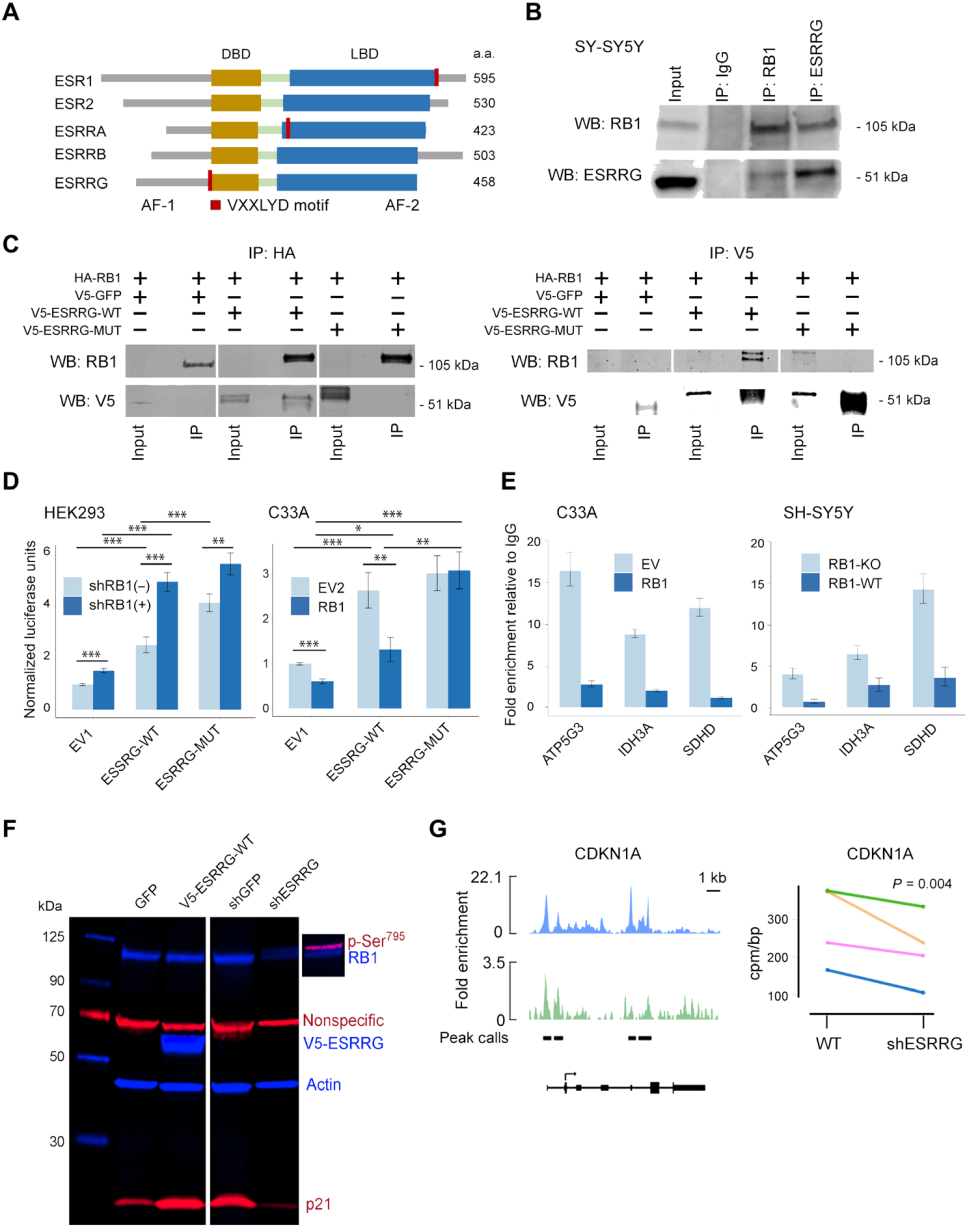
RB1 interacts with and inhibits ESRRG. (**A**) Protein maps showing location of RB1-binding VXXLYD motifs in ESRs (ESR1 and ESR2) and estrogen-related receptors (ESRRA, ESRRB, and ESRRG). AF, activation function domain; a.a., amino acids. (**B**) Western blot (WB) for endogenously expressed ESRRG or RB1 in RB1-WT SH-SY5Y neuroblastoma cells following immunoprecipitation for RB1, ESRRG or an immunoglobulin G (IgG) control. (**C**) Western blot for ectopically expressed hemagglutinin (HA)–tagged RB1 (HA-RB1), V5-tagged WT ESRRG (V5-ESRRG-WT), or ESRRG with mutated VXXLYD motif (V5-ESRRG-MUT) in RB1-null C33A cells following immunoprecipitation for HA (left). Western blot for ectopically expressed HA-RB1, V5-ESRRG-WT, or V5-ESRRG-MUT in RB1-null C33A cells following immunoprecipitation for V5 (right). IP, immunoprecipitation. (**D**) Normalized ERRE luciferase reporter activity with or without ectopic expression of V5-ESRRG-WT or V5-ESRRG-MUT as well as with (shRB1^+^) or without (shRB1^−^) doxycycline-induced RB1 knockdown in HEK293 cells (left, *n* = 12) or with (RB1) or without (EV2) RB1 re-expression in C33A cells (right, *n* = 12), respectively. Data are shown as means ± SEM. *P ≤ 0.05, **P ≤ 0.01, and ***P ≤ 0.001 (Student’s *t* test). (**E**) Chromatin immunoprecipitation for ESRRG followed by quantitative PCR in RB1-null C33A cells with and without exogenously expressed RB1 (*n* = 4) and in RB1-WT SH-SY5Y neuroblastoma cells with (RB1-KO) or without (RB1-WT) RB1-KO (*n* = 4). (**F**) Western blot in HEK293 cells following ectopic expression of V5-ESRRG-WT, shRNA directed against ESRRG (shESRRG), or controls (GFP and shGFP, respectively). (**G**) ESRRG ChIP-seq peaks at the *CDKN1A* locus and corresponding RNA-seq data with (shESRRG) and without (shGFP) ESRRG knockdown. DBD, DNA binding domain; EV, empty vector; LBD, ligand binding domain.

One of the frequently mutated genes implicated in the ESR/ESRR interaction network was *RB1* itself (Fig. 2A). ESR1, ESRRA, and ESRRG contain a VXXLYD motif, similar to the IXXLFD RB1-binding motif in E2F1 and E2F2 (Fig. 4A) (*15*–*18*). In coimmunoprecipitation experiments, we found that endogenous RB1 and ESRRG interact and that interaction of exogenously expressed RB1 and ESRRG is disrupted by alteration of the VXXLYD motif in ESRRG (Fig. 4, B and C). Among the three ESR/ESRR family members with a VXXLYD motif, only ESRRG contains this motif within its DNA binding domain (Fig. 4A), raising the possibility that RB1 inhibits ESRRG’s transcriptional activation by blocking its interaction with ERRE regulatory motifs (*19*). Consistent with this idea, the ectopic expression of wild-type ESRRG (V5-ESRRG-WT) activated a luciferase reporter downstream of the ERRE promoter, and this activation was enhanced by short hairpin RNA (shRNA)–mediated knockdown of RB1 in RB1-WT human embryonic kidney (HEK) 293 cells (Fig. 4D). Expression of an ESRRG mutant in which the VXXLYD motif was mutated (V5-ESRRG-MUT) resulted in greater activation of the reporter, which was only minimally enhanced by RB1 knockdown, indicating that loss of the RB1-ESRRG interaction results in constitutive ESRRG activity. Further supporting this finding, the ERRE luciferase reporter was activated by WT ESRRG in RB1-null C33A cells, and this activation was abrogated by ectopic expression of RB1. The RB1-binding mutant V5-ESRRG-MUT also activated the reporter, and it was resistant to inhibition by RB1. Moreover, we showed using ChIP–quantitative polymerase chain reaction (qPCR) that exogenous expression of RB1 in C33A cells decreased interaction of ESRRG with promoters of canonical ESRRG-regulated genes (*SDHD*, *IDH3A*, and *ATP5G3*) (*20*, *21*), whereas knockout (KO) of RB1 in RB1-WT SH-SY5Y neuroblastoma cells resulted in increased ESRRG interaction with these promoters (Fig. 4E and fig. S7). Consistent with previous work in prostate cancer cells (*22*), we found that ectopic expression of V5-ESRRG-WT in HEK293 cells induced p21 expression associated with hypophosphorylation (activation) of RB1, whereas knockdown of endogenous ESRRG resulted in depletion of p21 and hyperphosphorylation (inactivation) of RB1 (Fig. 4F). Similarly, in Rb cells, ESRRG was enriched at the *CDKN1A* gene (encoding p21), and knockdown of ESRRG decreased *CDKN1A* RNA expression, indicating direct transcriptional activation of *CDKN1A* by ESRRG (Fig. 4G). Thus, RB1 directly inhibits ESRRG, which, in turn, negatively regulates itself by activating p21, but these inhibitory mechanisms are lost in Rb. After *RB1*, the second most commonly mutated gene in Rb is the BCL6 corepressor (*BCOR*) (Fig. 1), and we found that BCOR represses ESRRG transcriptional activity independently of RB1 (fig. S8), suggesting that mutations in this gene also serve to increase ESRRG activity.

### ESRRG is a hypoxic adaptation and survival factor in Rb cells

**Fig. 5.**
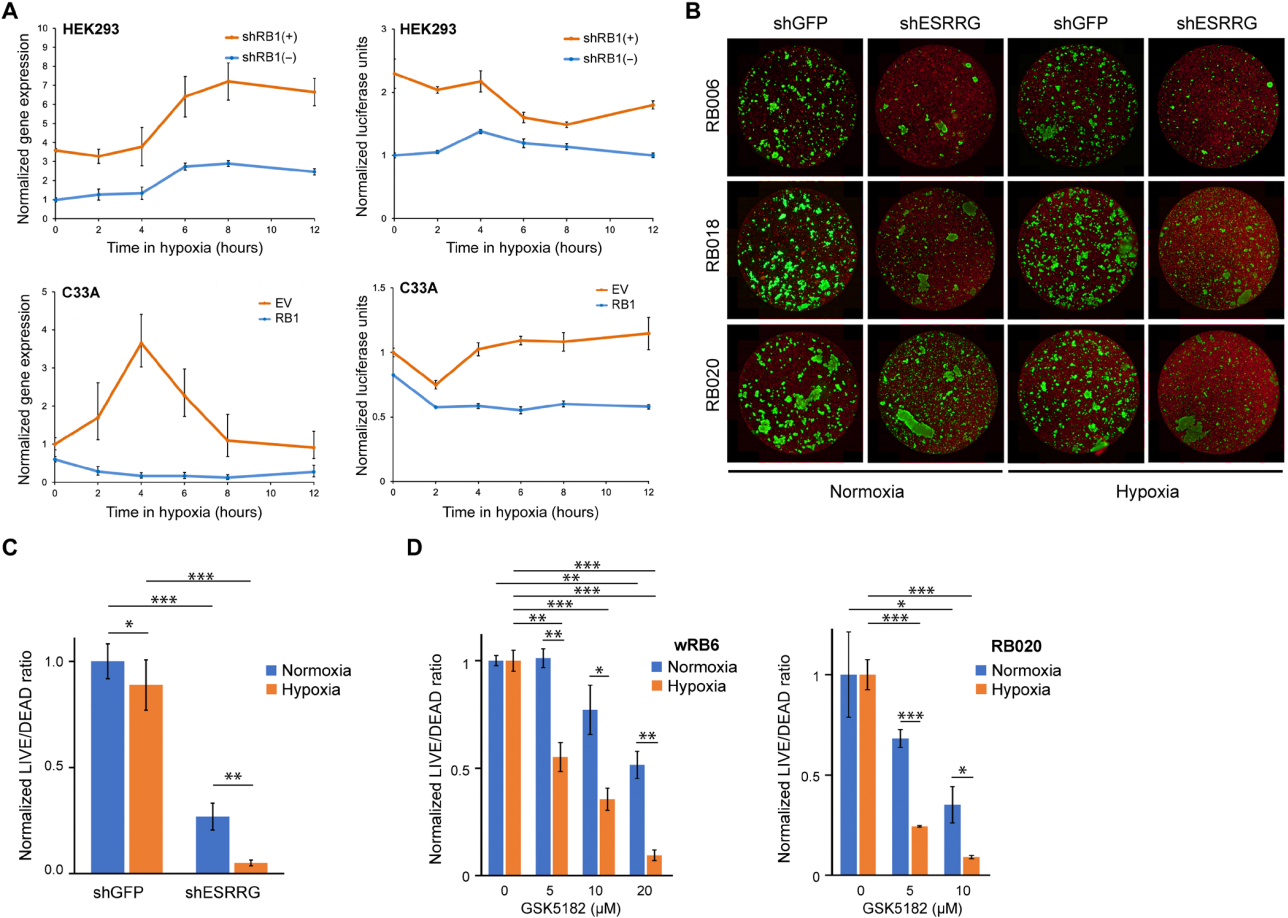
ESRRG is a hypoxic adaptation and survival factor in Rb. (**A**) Normalized gene expression of *ESRRG* (top left, *n* = 3) and normalized ERRE luciferase activity (top right, *n* = 6) in HEK293 cells with (shRB1^+^) or without (shRB1^−^) doxycycline treatment to induce shRNA-mediated knockdown of RB1 at the indicated time points in hypoxia (1% O_2_). Normalized gene expression of *ESRRG* (bottom left, *n* = 4) and normalized ERRE luciferase activity (bottom right, *n* = 3) in C33A cells with ectopically expressed RB1 (RB1) or EV control at the indicated time points in hypoxia (1% O_2_). (**B**) Representative images of LIVE (green)/DEAD (red) cell viability assays of three recently established Rb cell lines (RB006, RB018, and RB020) stably expressing shRNA directed against ESRRG (shESRRG) or GFP control (shGFP) cultured in normoxia versus hypoxia (1% O_2_) for 21 days. (**C**) Normalized LIVE/DEAD cell ratios averaged for all three Rb cell lines illustrated in (B) in normoxia versus hypoxia O_2_ (1%). *P ≤ 0.05, **P ≤ 0.01, and ***P ≤ 0.001 (Student’s *t* test). (**D**) Normalized LIVE/DEAD cell ratios in wRB6 cells (left, *n* = 3) and RB020 cells (right, *n* = 3) treated with the indicated micromolar concentrations of the ESRRG inverse agonist GSK5182 for 7 days in normoxia versus hypoxia (1% O_2_). *P ≤ 0.05, **P ≤ 0.01, and ***P ≤ 0.001 (Student’s *t* test). All data are shown as means ± SEM.

**Fig. 6.**
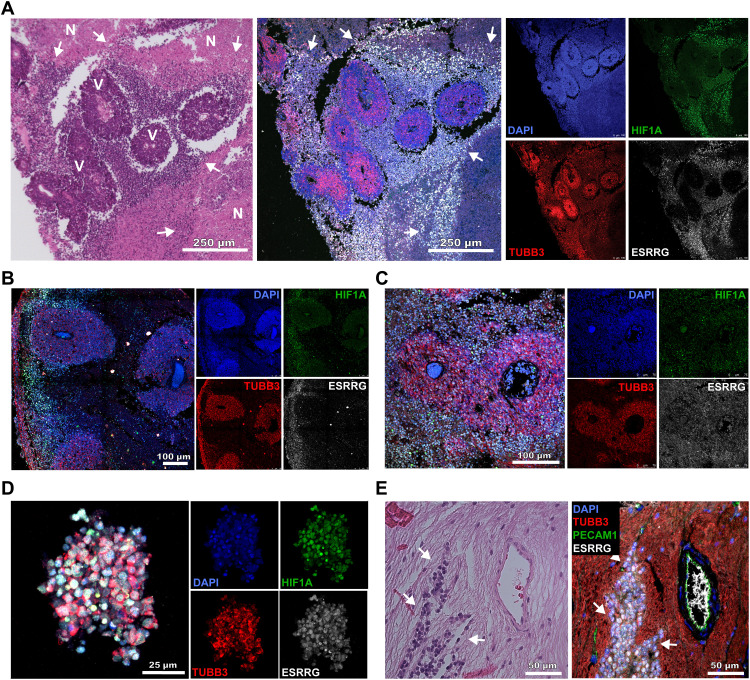
ESRRG expression in human Rbs. (**A**) Hematoxylin and eosin staining (left) and multiplex fluorescence immunohistochemistry (middle and right) of Rb sample #2587-18. ESRRG (white), TUBB3 (red), 4′,6-diamidino-2-phenylindole (DAPI) (blue), and HIF1A (green). V, viable tumor cells concentrated in cuffs around blood vessels; N, necrotic tumor cells beyond ~100 μm of tumor blood vessels. Arrows demarcate regions enriched for ESRRG- and HIF1A-expressing cells in hypoxic zones between viable and necrotic tumor cells. (**B** and **C**) Similar multiplex fluorescence immunohistochemistry findings in Rb samples #2647-18 (left panels) and #1109189582 (right panels). ESRRG (white), TUBB3 (red), DAPI (blue), and HIF1A (green). (**D**) Vitreous tumor cell cluster or “seed” in Rb sample #2647-18 demonstrating strong diffuse staining for ESRRG and HIF1A. ESRRG (white), TUBB3 (red), DAPI (blue), and HIF1A (green). (**E**) Hematoxylin and eosin staining (left) and multiplex fluorescence immunohistochemistry (right) in Rb sample #29-15 showing invasion of the optic nerve by tumor cells (arrows). ESRRG (white), TUBB3 (red), DAPI (blue), and PECAM1 (green).

Given the known role of ESRRG in hypoxic adaptation in the retina and other tissues (*23*, *24*), we investigated how RB1 loss affects regulation of ESRRG under hypoxic conditions. In RB1-WT HEK293 cells, hypoxic conditions (1% O_2_) induced a transient ~3-fold increase in *ESRRG* mRNA expression, which was associated with transient modest activation of the ERRE luciferase reporter (Fig. 5A). In HEK293 cells depleted of RB1, *ESRRG* expression levels were more than threefold higher at baseline and demonstrated an exaggerated increase in response to hypoxia, which was accompanied by hyperactivity of the ERRE reporter over the same time course. Conversely, RB1-null C33A cells demonstrated an exaggerated response to hypoxia in terms of both *ESRRG* expression and ERRE reporter activity, both of which were repressed by ectopic expression of RB1 (Fig. 5A). In Rb cell lines, shRNA-mediated depletion of ESRRG or inhibition of ESRRG using the specific inverse agonist GSK5182 (*25*) resulted in marked cell death, which was exacerbated in hypoxia (Fig. 5, B to D). Together, these findings suggest that RB1 dampens the hypoxia-induced surge in ESRRG activity and that loss of RB1 abolishes this homeostatic mechanism, resulting in nascent Rb cells becoming increasingly dependent on ESRRG in the hypoxic tumor microenvironment. Consistent with this possibility, ESRRG is normally expressed only in isolated retinal cells (fig. S9), but it is diffusely expressed in Rb tumors, particularly in hypoxic regions beyond ~100 μm of tumor blood vessels (Fig. 6A) and in hypoxic vitreous “seeds” (Fig. 6B). Intense ESRRG expression was also observed in Rb cells invading the optic nerve (Fig. 6C), which is strongly associated with metastasis and poor outcome (*26*).

## DISCUSSION

In this study, we show that (i) recurrent mutations in Rb tumors may have in common the effect of increasing ESRRG activity, (ii) RB1 interacts with and inhibits ESRRG such that mutational inactivation of RB1 may release ESRRG from negative regulation and confer upon it oncogenic potential, and (iii) Rb cells are dependent on ESRRG for survival and hypoxic adaptation, which may create a selective pressure for acquiring genomic aberrations that increase ESRRG activity. The most commonly mutated gene in Rb, after *RB1* itself, is the transcriptional corepressor *BCOR*, a component of the noncanonical polycomb repressive complex (PRC) 1.1 that is also mutated in other developmental disorders affecting the eye (*27*, *28*). Inactivating mutations in *BCOR* are found in about 20% of Rb tumors and are associated with poor prognosis (*10*, *29*). We show here that BCOR represses ERRE-mediated transcription independently of RB1, suggesting that BCOR loss derepresses ESRRG beyond that associated with RB1 loss. Because *ESRRG* is located on chromosome 1q, the most common chromosome copy number aberration in Rb, and as we found examples of more complex genomic rearrangements affecting the *ESRRG* locus, it is interesting to speculate that chromosome-level alterations may also contribute to increased ESRRG activity in Rb. Several other genes located on 1q have also been implicated in Rb progression (*5*, *30*), suggesting that 1q gain may promote tumor progression through its effect on multiple genes. Together, recurrent mutations in Rb may have in common the effect of increasing ESRRG activity, which could make ESRRG an attractive therapeutic target.

ESRRG is an estrogen-related orphan nuclear receptor transcription factor that is normally expressed in the developing retina and central nervous system, where it regulates genes involved in development, proliferation, and oxygen metabolism (*23*, *24*, *31*–*33*). In Rb cells, we found that ESRRG localizes to and regulates the transcription of numerous genes containing CRX, OTX2, NEUROD, and LHX binding motifs that regulate normal retinal development (*34*–*36*). CRX and OTX2 in particular are key regulators of oxidative metabolism and mitochondrial function during early neurogenesis and retinogenesis (*37*–*39*), and they can become oncogenic when aberrantly activated (*40*, *41*). The developing retina is a hypoxic environment owing to the proliferation of retinal precursor cells, oxygen demand of newly formed neurons, and limited blood supply (*42*, *43*). This oxygen demand is markedly increased in Rb due to uncontrolled proliferation of tumor cells (*44*, *45*). We showed that RB1 loss increases promoter occupation of ESRRG at genes involved in oxidative metabolism and that Rb cells require ESRRG for survival, proliferation, and hypoxic adaptation. Rb cells demonstrate constitutive ESRRG expression that is enriched in oxygen-poor regions beyond ~100 μm from tumor blood vessels (*46*) and in vitreous seeds, which consist of clusters of Rb cells that proliferate in the hypoxic vitreous cavity and portend chemoresistance and poor outcome in Rb (*47*, *48*). Further, ESRRG expression clearly demarcates aggressive Rb cells invading the optic nerve, which is a major risk factor for metastatic death (*26*).

In conclusion, Rb tumors undergo ongoing hypoxic adaptation to allow continued proliferation and invasion in an increasingly oxygen-depleted environment (*49*, *50*), and our findings implicate ESRRG and its decoupling from normal homeostatic regulation by RB1 and BCOR as a key mediator of this hypoxic adaptation. Further studies are warranted to investigate the potential role for inhibiting ESRRG in the management of Rb.

## MATERIALS AND METHODS

### Specimens and clinical data

Human Rb samples were collected from enucleated eyes at the time of surgery, snap-frozen, and stored at −80°C until used for analysis with the approval from the University of Miami Institutional Review Board (no. 20130636). Written informed consent was obtained from each patient. Clinical and histopathologic information were obtained, and samples were deidentified for further analysis. All data were included in the analysis.

### DNA sequencing, quality control, and alignment

WES was performed on 31 of our Rb enucleated tumor samples, 20 of which had matched blood DNA available for sequencing. DNA was extracted using the Wizard Genomic DNA Purification Kit (Promega, Madison, WI) and the QuickGene DNA Whole Blood Kit S (Fujifilm, Tokyo, Japan), respectively. Exome fragments were captured using the SureSelect Human Exon V5 (Agilent) and sequenced on the Illumina HiSeq 2500. WES data from an additional 72 Rb samples with matched blood were obtained from the European Genome-Phenome Archive (EGAS00001001690) with permission from the Data Access Committee (EGAC00001000431) (*7*). WGS from four Rb samples with matched blood was obtained with permission from dbGaP (phs000352.v1.p1). FASTQ files from all sources underwent the following bioinformatics pipeline. Sequence data were quality-controlled using FastQC (v0.11.3). WES and WGS reads were trimmed (if required) and aligned to the human genome (hg19/GRCH37) using NovoAlign (v3.06.04), marked for duplicates using Picard (v1.128), and realigned around small and large indels using Abra (v0.94c) (*51*), and read mate was fixed and analyzed for coverage statistics using Picard.

### Mutation analysis

WES datasets underwent variant calling for single-nucleotide polymorphisms (SNPs) and indels using MuTect2 (GATK 2016-01-25 nightly build) (*52*). For MuTect2, mutation calls for a panel of normals (*n* = 117 germline blood samples) were generated and pooled together to further filter out mutations that were present in at least two normals. For tumors without matched blood samples, MuTect2 was used for variant calling with a panel of normals and a high coverage blood sample to filter out likely germline mutations. Mutations were further filtered out if alternate tumor read counts were <3, if alternate tumor read counts were <8 and blood alternate read counts were >1, and if the minor allele frequency (MAF) was <5%. For all sequencing samples, the BAM files were investigated manually for the regions of interest (*RB1* and *BCOR*) using the Integrative Genomics Viewer (v2.3.80, Broad Institute, Cambridge, MA).

For all called mutations, ANNOVAR was used for annotation (*53*). Following annotation, mutations were filtered out if the MAF was 1% or greater in the population according to 1000 Genomes Project (August 2015) or Exome Sequencing Project (March 2015), and mutations listed in Database of Single Nucleotide Polymorphisms (dbSNP) (v138) were filtered out, with the exception of SNPs with a MAF < 1% (or unknown) in the population, a single mapping to reference assembly, or with a “clinically associated” tag. If the exact same insertion or deletion or non-frameshift multinucleotide substitution was present in at least two samples without matched germ line and were not present in any matched tumor sample, it was removed as a likely germline variant. Functional consequences of SNPs were assessed by three mutational predictor tools: PolyPhen-2 (probably damaging, possibly damaging, benign) (*54*), Functional Analysis through Hidden Markov Models (FATHMM) (damaging, tolerated) (*55*), and MetaLR (damaging, tolerated) (*56*). In nonexonic regions and for synonymous mutations, SNPs were considered deleterious if two of three of the above prediction algorithms predicted a damaging mutation. For nonsynonymous exonic SNPs, mutations were not considered deleterious if two of three algorithms predicted a benign or tolerated mutation. Probably damaging, possibly damaging, or damaging calls were considered deleterious. All insertions and deletions in exonic regions and alterations in splicing junctions were considered deleterious. For identification of additional driver mutations, the unmatched tumor samples were first excluded before being evaluated if mutations were found in matched samples and were also excluded if known to be one of the benign mutations commonly seen in cancer exomes (*57*, *58*). Lollipop plots displaying the distribution of driver mutations along the protein domains of each gene were plotted using trackViewer (*59*) in R (version 4.0.3).

### Copy number variations

CNVs were ascertained from WES data using CNVkit (v0.8.5) (*60*) and by cgpBattenberg using default settings (*61*). Isodisomy was determined by plotting B allele frequencies using CNVkit, Allele-Specific Copy Number Analysis of Tumors (ASCAT), and cgpBattenberg. CNVkit output was analyzed using Genomic Identification of Significant Targets in Cancer (GISTIC) (*62*) to determine significant CNVs using cutoffs of FDR < 0.05, *z* score ≥ 6, and chromosomal arm score > |0.1|. *MYCN* amplification was determined using GISTIC with a *z* score cutoff of 3. Structural variants from the WGS samples were determined using DELLY (version 0.7.6) (*63*).

### Pathway analysis

Genes with mutations in at least two Rb samples were loaded into the “Path Designer” tool of the Ingenuity Pathway Analysis product (QIAGEN, Valencia, CA, USA), and an unsupervised interaction network identifying additional interacting members was generated using the “path explorer” option in the “build” workbox, restricting the software’s analysis output to paths of documented interaction with a maximum of one intermediary node between the selected proteins.

### Cell culture

Newly established, patient-derived low-passage Rb cell lines (wRB6, RB006, RB010, RB015, RB016, RB018, RB020, and RB021) were derived from enucleated eyes of patients of the senior author (J.W.H.) and cultured at 5% O_2_ in Dulbecco’s modified essential medium (DMEM)/F12 with B-27 minus vitamin A (Life Technologies), 1% penicillin-streptomycin, 2 mM GlutaMAX (Gibco), basic fibroblast growth factor (10 ng/ml; PeproTech, catalog no. 100-18B), recombinant human stem cell factor (10 ng/ml; PeproTech, catalog no. 300-07), and epidermal growth factor (20 ng/ml; PeproTech, catalog no. AF-100-15). All low-passage Rb cell lines had an average doubling time of ~72 hours and a viability of ~80%. Human C33A cells were obtained from the American Type Culture Collection (ATCC) and maintained in Eagle’s minimum essential medium supplemented with 10% heat-inactivated fetal bovine serum (HI-FBS) and 1% penicillin-streptomycin. Human HEK293 cells were obtained from ATCC and maintained in DMEM with 10% HI-FBS, 2 mM GlutaMAX, and 1% penicillin-streptomycin. Of note, although RB1-WT HEK293 cells express adenoviral E1A that partially inhibits RB1, it does not affect the interaction of RB1 with E2F1 (*64*) or with ESRRG (fig. S10). RB1-WT neuroblastoma cell line (SH-SY5Y) was generously provided by S. Dhar’s laboratory at the University of Miami. SH-SY5Y cells were cultured in DMEM/F12 (1:1) supplemented with 10% HI-FBS. Cell proliferation and viability were measured using Trypan Blue Stain (Gibco) and/or LIVE/DEAD Cell Viability Assay (Thermo Fisher Scientific). All cells were maintained in ambient O_2_ unless otherwise stated. Rb cells were treated with various concentrations of the ESRRG selective inverse agonist GSK5182 (Aobious Inc., Gloucester, MA) for 7 days.

### Plasmids and lentivirus expression vectors

shRNA plasmids were created by integrating validated small interfering RNA (siRNA) sequences 5′-CCTGTCAGGAAACTGTATGAT-3′ (*65*) and 5′-AATGGCCATCAGAACGGACTT-3′ targeting human *ESRRG* (shESRRG) into pLKO.1-Puro vector (Addgene, #8453). Nonspecific siRNA sequence 5′-AACAGCCACAACGTCTATATC-3′ or siRNA sequence 5′-CAACAGCCACAACGTCTATAT-3′ targeting green fluorescent protein (*GFP*) was used as controls for shRNA. Tetracycline-inducible TET-shRB1 and constitutive shRB1 constructs were created by integrating validated siRNA sequences 5′-GAAAGGACATGTGAACTTA-3′ (*66*) and 5′-GAACGATTATCCATTCAAA-3′ (*67*) targeting human *RB1* (shRB1) into TET-pLKO.1-Puro vector (Addgene #21915) and pLKO.1-Puro vector, respectively. The plasmids were packaged into lentiviral particles by transient cotransfection into HEK293T cells with pMD2G and psPAX2 packaging plasmids using jetPRIME reagent (Polyplus). The pCMV–ESRRG-WT-V5 vector was created by PCR amplification of *ESRRG* full-length cDNA fragment (Horizon, #MHS6278-202806209) and subsequent recombination into a pCMV plasmid encoding C-terminal V5. An RB1-binding site mutant of *ESRRG* (V5-ESRRG-MUT) was generated using site-directed mutagenesis to replace 5′-GTCAGGAAACTGTATGATGAC-3′ with 5′-GACAAAGTCGATGCGAGGTAT-3′. A construct encoding *RB1* was created by PCR-amplifying cDNA for human *RB1* (Horizon, #MHS6278-202758572) and cloning into an N-terminal hemagglutinin (HA)–tagged expression vector. SH-SY5Y cells with RB1-KO were created by transducing viruses encoding the CRISPR-Cas9 with guide RNAs targeting *RB1* (5′-CCGAAAAACGGCCGCCACCGC-3′ and 5′-CCGCGGAACCCCCGGCACCGC-3′). The transduced cells were selected with hygromycin and puromycin and clonally selected for RB1-KO. The pLL-FLAG-BCOR plasmid was a gift from V. Bardwell. The ERRE promoter luciferase reporter plasmid 3xERRE-luciferase (pERRE-LUC; Addgene, # 37851) was a gift from R. Riggins. CMV promoter–driven beta-galactosidase plasmid (pCMV-beta-gal) was obtained from ATCC (#77177). Lentiviral particles were concentrated by precipitation from culture supernatants using PEG-it (System Biosciences) and quantified with the QuickTiter Lentivirus Titer Kit (Cell Bio Labs). Gene knockdown and overexpression were verified with Western blot or qPCR using appropriate controls (Fig. 4 and figs. S10 and S11).

### Luciferase assays

RB1-null C33A cells were cotransfected with indicated quantities of pCMV-HA-RB1, pLL-FLAG-BCOR, pCMV-V5-ESRRG-WT or pCMV-V5-ESRRG-MUT, and pERRE-LUC using jetPRIME reagent (Polyplus). Stoichiometric quantities of empty vectors pCMV-V5-EV, pCMV-HA-EV, and pLL-FLAG-EV were used for C33A cell transfection normalization. HEK293 cells expressing TET-shRB1 (HEK293-TET-shRB1) were induced with doxycycline for 48 hours and then cotransfected with indicated quantities of pCMV-HA-RB1, pCMV-V5-ESRRG-WT or pCMV-V5-ESRRG-MUT, and pERRE-LUC. Cells not treated with doxycycline and stoichiometric quantities of EVs pCMV-V5-EV and pLL-FLAG-EV were used for HEK293 cell transfection normalization. Luciferase activity was measured 24 hours after plasmid transduction by lysing the cells in the passive buffer (Promega) and combining equal volumes of cell lysates with the Bright-Glo reagent (Promega). Cotransfection with a CMV-beta-gal plasmid and bicinchoninic acid protein quantification assays were used for luciferase signal normalization.

### Coimmunoprecipitations

RB1-null C33A and RB1-WT HEK293 cells (10^7^ cells per condition) and SH-SY5Y neuroblastoma cells (20^7^ cells per condition) were cultured in 15-cm dishes, transfected with the indicated expression plasmids, and lysed with ice-cold NP-40 buffer supplemented with protease and phosphatase inhibitors (Roche). All cells were treated with dithiobis succinimidyl propionate (Thermo Fisher Scientific) directly on plastic. Cell lysates were passed 20 times through a 25-gauge needle, incubated on ice for 1 hour, pelleted by centrifugation at 12,000 relative centrifugal force (RCF) for 10 min, precleared by 30 min coincubation with magnetic beads (Life Technologies) at 4°C, and incubated overnight at 4°C with 5 μg of the indicated antibodies per 1 mg of quantified protein lysates. Antibody-protein conjugates were captured by incubation with protein G Dynabeads for 1 hour, washed with ice-cold coimmunoprecipitation buffer, and eluted by boiling in SDS running buffer. Total precleared lysates were used as positive control. Antibodies used for these experiments are listed in data S4.

### Western blot

Cell pellets were resuspended in lysis buffer, which consisted of 50 mM Hepes (pH 7.2), 400 mM NaCl, 0.1% NP-40, 0.5 mM EDTA (pH 8), 2.5 mM dithiothreitol plus protease, and phosphatase inhibitors. Resulting cell lysates were incubated on ice for 10 min, sonicated at medium power for 10 s, centrifuged for 10 min at 10,000 RCF to remove cellular debris, and quantified, and equal quantities were denatured by boiling with SDS loading buffer for 5 min at 90°C. Proteins (30 μg per condition) were resolved on SDS–polyacrylamide gel electrophoresis gradient gels and transferred to a polyvinylidene difluoride membrane (Thermo Fisher Scientific). Blots were blocked in 5% bovine serum albumin (BSA) in tris-buffered saline (TBS; pH 7.6), probed overnight with indicated primary antibodies in 5% BSA and 0.15% Tween 20 in TBS (TBS-T), washed in TBS-T, and incubated for 1 hour with a secondary antibody in 5% BSA/TBS-T solution. Proteins were visualized using SuperSignal chemiluminescence (Thermo Fisher Scientific) or LI-COR imaging technology (LI-COR Biosciences). Antibodies used for these experiments are listed in data S4.

### Hypoxia experiments

Recently established Rb cell lines (RB006, RB018, RB020, and RB021) were transduced with either shESRRG or shCTRL lentiviral particles and treated with puromycin (2 μg/ml) 48 hours after transduction to select for stable lentiviral integration. At 7 days after transduction, cells were maintained in normoxia or hypoxia (1% O_2_). Cell viability at day 21 was measured with the LIVE/DEAD Cell Viability Assay (Thermo Fisher Scientific). Whole wells were scanned using the EVOS FL Auto Imaging System (Thermo Fisher Scientific), and image fluorescence was quantified using particle analysis with ImageJ software. HEK293 cells stably integrated with TET-shRB1 were induced with doxycycline hyclate (1 μg/ml) for 48 hours, transfected with pERRE-LUC plasmid, and maintained in either normoxia or hypoxia (1% O_2_) for an additional 24 hours. C33A cells were transfected with pCMV-HA-RB1 and pERRE-LUC plasmids for 24 hours and exposed to either normoxia or hypoxia (1% O_2_) for 24 hours. Uninduced HEK293-TET-shRB1 and C33A cells transfected with pCMV-HA-EV were used as experimental controls.

### Quantitative real-time PCR

Total RNA was extracted from cells with a combination of TRIzol and an RNeasy Mini RNA isolation kit (QIAGEN, Valencia, CA, USA) and reverse-transcribed using a high-capacity cDNA transcription kit (Applied Biosystems, Foster City, CA, USA). qPCR was performed in triplicate using the 7300 Real-time reverse transcription polymerase chain reaction (RT-PCR) system (Applied Biosystems) according to the manufacturer’s description using the following thermocycler parameters: 1 min of initial activation at 98°C, 40 cycles of 5-s denaturation at 98°C, and 55-s annealing and extension at 60°C. The relative gene expression data were analyzed by the comparative CT (ΔΔCT) method. The results were normalized to *GAPDH*, *RNA18S*, and *ACTB* as internal controls. Oligonucleotide primer sequences used are listed in data S4.

### ChIP sequencing and DNA sequencing

Recently established low-passage RB006 and RB018 cell lines with 1q gain, C33A cells with and without exogenous RB1 expression, and SH-SY5Y neuroblastoma cells with and without RB1-KO were grown to approximately 5 × 10^7^ cells, collected, and pelleted. Cell pellets were fixed in 1% formaldehyde for 10 min to cross-link protein-DNA interactions. Cell nuclei were extracted and subjected to sonication in a Bioruptor Pico (Diagenode). Shearing was optimized to yield chromatin fragments of 200 to 500 base pairs. Chromatin was incubated in ChIP-qualified antibody against ESRRG (data S4). Antibodies were captured using protein G–coupled magnetic beads, and protein-DNA was eluted. DNA was purified for downstream analysis. Successful ChIP was validated with RT-PCR for known binding genes and known negative controls. Technical controls included nonspecific immunoglobulin G incubation of cell lysates and no-antibody (input) controls for nonspecific DNA binding to protein G–coupled beads. DNA was sent for library preparation and sequencing in the Oncogenomics Shared Resource of the University of Miami. Bioinformatic analysis was performed by established ChIP-seq pipelines including quality control (FastQC), adapter trimming and alignment (NovoAlign), and normalization to an input control followed by peak calling (MACS2). Heatmap and band plots were generated using deepTools. Overlapping high-confidence peaks that were called in both the RB006 and RB018 ESRRG pulldown ChIP-seq datasets as well as a pooled dataset that combined the RB006 and RB018 data were determined using HOMER (*P* < 0.001) and used for downstream analyses. Euler diagram of peaks called for each dataset and overlapping datasets was plotted using eulerAPE (version 3.0) (*68*). Motif and gene set enrichment analyses (GSEAs) were conducted using HOMER. Super-enhancer regions identified in retinal and neural tissue were downloaded from the comprehensive human super-enhancer database (version 1.03) (*14*). Peaks with pooled peak calls, super-enhancer locations, exon and intron locations, and direction of transcription were plotted for visualization using our custom program SparK (version 2.6.2) (*69*). Peak location, motif analysis, and co-occurrence visualization were conducted using ChIPseeker, UpSetR, and circlize in R (*70*–*72*). ChIP-qPCR primer sequences are listed in data S4.

### RNA-seq and analysis

RNA was extracted from tumor samples or Rb cell lines (RB006, RB016, RB018, and wRB6) with or without shESRRG knockdown using a combination of TRIzol and an RNeasy Mini RNA isolation kit (QIAGEN, Valencia, CA, USA). RNA-seq libraries were prepared using the TruSeq Stranded Total RNA Prep Kit with Ribo-Zero Gold to remove cytoplasmic and mitochondrial ribosomal RNA according to the manufacturer’s recommendation (Illumina, San Diego, CA). Total RNA-seq libraries were run on an Illumina NextSeq 500 sequencing instrument according to the manufacturer’s protocol. RNA-seq from the four Rb samples that underwent WGS was obtained with permission from dbGaP (phs000352.v1.p1). Reads were aligned and gene counts were made using STAR, data quality was assessed using FastQC and RSeQC, and gene expression was normalized, batch-corrected, and determined using EdgeR. Significant differences (FDR < 0.05) between cells expressing shESRRG versus shGFP control were calculated using EdgeR. For tumor samples that underwent RNA-seq without a detectable biallelic loss of RB1 seen in exome analysis, RNA tracks were manually curated to look for any *RB1* mutations.

### Integration of ChIP-seq and RNA-seq data

Significant ESRRG ChIP-seq peaks overlapping among RB006, RB018, and pooled samples were correlated with significantly up-regulated or down-regulated genes after shESRRG knockdown in four primary cultured cell lines (RB006, RB016, RB018, and wRB6). Genes that were significantly up-regulated or down-regulated in association with ESRRG ChIP-seq peaks were analyzed with GSEA and MSigDB. Differentially expressed genes after ESRRG knockdown were evaluated for ESRRG-associated transcription factor motifs.

### scRNA-seq analysis

Single-cell suspensions were counted using the Cellometer K2 Fluorescent Viability Cell Counter (Nexcelom), verified manually using a hemocytometer, and adjusted to 1000 cells/μl. For RNA-seq, samples were prepared using the Chromium Single Cell 5′ Library & Gel Bead Kit v2 (10X Genomics) according to the manufacturer’s protocol with a capture target of 10,000 cells. Each sample was processed on an independent Chromium Single Cell A Chip (10X Genomics) and run on a thermocycler (Eppendorf). 5′ gene expression libraries were sequenced using NextSeq 500 150-cycle high-output flow cells or NovaSeq 6000 200-cycle S4 flow cells. Raw base call (BCL) files were analyzed using CellRanger (version 3.0.2). The “mkfastq” command was used to generate FASTQ files, and the “count” command was used to generate raw gene barcode matrices aligned to the GRCh38 Ensembl build 93 genome. The data from all five samples were combined in R (3.5.2) using the Seurat package (3.0.0), and an aggregate Seurat object was generated (*73*, *74*). To ensure our analysis was on high-quality cells, filtering was conducted by retaining cells that had unique molecular identifiers greater than 400, expressed 100 to 6000 genes inclusive, and had mitochondrial content less than 10%. This resulted in 6371 cells for RB025, 12,808 cells for RB026, 10,675 cells for RB027, 1354 cells for RB028, and 1445 cells for RB029. Doublets were assessed using the DoubletFinder (2.0.3) algorithm (*75*) using the multiplet rates in the 10X Genomics Chromium Single Cell 3′ Reagent Kits User Guide (v3 Chemistry). Doublets were assessed by individual samples based on the number of captured cells. Data for all five samples were combined using the Standard Integration Workflow (https://satijalab.org/seurat/v3.0/integration.html). Data from each sample were normalized using the NormalizeData() function, and variable features were identified using FindVariableFeatures() with 5000 genes and the selection method set to “vst,” a variance stabilizing transformation. To identify integration anchor genes among the five samples, the FindIntegrationAnchors() function was used with 30 principal components (PCs) and 5000 genes. Using Seurat’s IntegrateData(), the samples were combined into one object. The data were scaled using the ScaleData() function to reduce the dimensionality of this dataset. Principal components analysis (PCA) was performed, and the first 30 PCs were summarized using uniform manifold approximation and projection (UMAP) dimensionality reduction (*76*). Thirty PCs were chosen on the basis of results from analysis using JackStraw() and elbow plots. The DimPlot() function was used to generate the UMAP plots (Fig. 2C). Clustering was conducted using the FindNeighbors() and FindClusters() functions using 30 PCA components and a resolution parameter set to 1.8. The original Louvain algorithm was used for modularity optimization (*77*). The resulting 36 louvain clusters were visualized in a two-dimensional UMAP representation. Differentially expressed genes were identified for each cluster using FindAllMarkers() and filtered for |log fold change| greater than 0.5 and adjusted *P* value less than 0.05. Dot plots were generated using the DotPlot() function, violin plots were generated with the vlnplot() function, and scatterplots were generated with FeatureScatter() in Seurat with “assay” set to “RNA.”

### Immunohistochemistry

For immunohistochemistry, 4-μm paraffin sections of Rb tumor specimens were deparaffinized in xylene, rehydrated in an ethanol gradient, permeabilized for 30 min in 0.5% Triton X-100 (v/v in phosphate-buffered saline), and subjected to antigen retrieval using sodium citrate buffer (pH 6.0) at 95°C for 20 min. Sections were then washed, equilibrated in water for 1 hour, and blocked with 1% BSA for 1 hour at room temperature. Samples were then incubated in primary antibody overnight at 4°C, washed 3× in distilled water, and then incubated in secondary antibody for 2 hours at room temperature. Samples were then washed 3× in distilled water, mounted with SlowFade Diamond Antifade mounting medium with 4′,6-diamidino-2-phenylindole (Thermo Fisher Scientific), and imaged using an SP8 Leica laser scanning confocal microscope (Leica Microsystems Inc., Buffalo Grove, IL).

### Statistical analyses

Data statistics and bioinformatics analyses were performed using R (version 4.0.3) and Bioconductor packages (www.bioconductor.org/) unless otherwise indicated. Multiple comparisons were adjusted by the Benjamini-Hochberg correction.

## Notes

View/request a protocol for this paper from *Bio-protocol*.

## References

[1] Global Retinoblastoma Study Group, I. D. Fabian, E. Abdallah, S. U. Abdullahi, R. A. Abdulqader, S. A. Boubacar, D. S. Ademola-Popoola, A. Adio, A. R. Afshar, P. Aggarwal, A. E. Aghaji, A. Ahmad, M. N. R. Akib, L. A. Harby, M. H. Al Ani, A. Alakbarova, S. A. Portabella, S. A. F. Al-Badri, A. P. A. Alcasabas, S. A. Al-Dahmash, A. Alejos, E. Alemany-Rubio, A. I. A. Bio, Y. A. Carreras, C. Al-Haddad, H. H. Y. Al-Hussaini, A. M. Ali, D. B. Alia, M. F. Al-Jadiry, U. Al-Jumaily, H. M. Alkatan, C. All-Eriksson, A. A. R. M. Al-Mafrachi, A. A. Almeida, K. M. Alsawidi, A. A. S. M. Al-Shaheen, E. H. Al-Shammary, P. O. Amiruddin, R. Antonino, N. J. Astbury, H. T. Atalay, L.-O. Atchaneeyasakul, R. Atsiaya, T. Attaseth, T. H. Aung, S. Ayala, B. Baizakova, J. Balaguer, R. Balayeva, W. Balwierz, H. Barranco, C. Bascaran, M. B. Popovic, R. Benavides, S. Benmiloud, N. B. Guebessi, R. C. Berete, J. L. Berry, A. Bhaduri, S. Bhat, S. J. Biddulph, E. M. Biewald, N. Bobrova, M. Boehme, H. C. Boldt, M. T. B. C. Bonanomi, N. Bornfeld, G. C. Bouda, H. Bouguila, A. Boumedane, R. C. Brennan, B. G. Brichard, J. Buaboonnam, P. Calderón-Sotelo, D. A. C. Jara, J. E. Camuglia, M. R. Cano, M. Capra, N. Cassoux, G. Castela, L. Castillo, J. Català-Mora, G. L. Chantada, S. Chaudhry, S. S. Chaugule, A. Chauhan, B. Chawla, V. S. Chernodrinska, F. S. Chiwanga, T. Chuluunbat, K. Cieslik, R. L. Cockcroft, C. Comsa, Z. M. Correa, M. G. C. Llano, T. W. Corson, K. E. Cowan-Lyn, M. Csóka, X. Cui, I. V. Da Gama, W. Dangboon, A. Das, S. Das, J. M. Davanzo, A. Davidson, P. De Potter, K. Q. Delgado, H. Demirci, L. Desjardins, R. Y. D. Coronado, H. Dimaras, A. J. Dodgshun, C. Donaldson, C. R. D. Macedo, M. D. Dragomir, Y. Du, M. D. Bruyn, K. S. Edison, I. W. E. Sutyawan, A. E. Kettani, A. M. Elbahi, J. E. Elder, D. Elgalaly, A. M. Elhaddad, M. M. A. Elhassan, M. M. Elzembely, V. A. Essuman, T. G. A. Evina, Z. Fadoo, A. C. Fandiño, M. Faranoush, O. Fasina, D. D. P. G. Fernández, A. Fernández-Teijeiro, A. Foster, S. Frenkel, L. D. Fu, S. L. Fuentes-Alabi, B. L. Gallie, M. Gandiwa, J. L. Garcia, D. G. Aldana, P. Y. Gassant, J. A. Geel, F. Ghassemi, A. V. Girón, Z. Gizachew, M. A. Goenz, A. S. Gold, M. Goldberg-Lavid, G. A. Gole, N. Gomel, E. Gonzalez, G. G. Perez, L. González-Rodríguez, H. N. G. Pacheco, J. Graells, L. Green, P. A. Gregersen, N. D. A. K. Grigorovski, K. M. Guedenon, D. S. Gunasekera, A. K. Gündüz, H. Gupta, S. Gupta, T. Hadjistilianou, P. Hamel, S. A. Hamid, N. Hamzah, E. D. Hansen, J. W. Harbour, M. E. Hartnett, M. Hasanreisoglu, S. Hassan, S. Hassan, S. Hederova, J. Hernandez, L. M. C. Hernandez, L. Hessissen, D. F. Hordofa, L. C. Huang, G. B. Hubbard, M. Hummlen, K. Husakova, A. N. Hussein Al-Janabi, R. Ida, V. R. Ilic, V. Jairaj, I. Jeeva, H. Jenkinson, X. Ji, D. H. Jo, K. P. Johnson, W. J. Johnson, M. M. Jones, T. B. A. Kabesha, R. L. Kabore, S. Kaliki, A. Kalinaki, M. Kantar, L.-Y. Kao, T. Kardava, R. Kebudi, T. Kepak, N. Keren-Froim, Z. J. Khan, H. A. Khaqan, P. Khauv, W. J. Kheir, V. Khetan, A. Khodabande, Z. Khotenashvili, J. W. Kim, J. H. Kim, H. Kiratli, T. T. Kivelä, A. Klett, J. E. K. K. Palet, D. Krivaitiene, M. Kruger, K. Kulvichit, M. W. Kuntorini, A. Kyara, E. S. Lachmann, C. P. S. Lam, G. C. Lam, S. A. Larson, S. Latinovic, K. D. Laurenti, B. H. A. Le, K. Lecuona, A. A. Leverant, C. Li, B. Limbu, Q. B. Long, J. P. López, R. M. Lukamba, L. Lumbroso, S. Luna-Fineman, D. Lutfi, L. Lysytsia, G. N. Magrath, A. Mahajan, A. R. Majeed, E. Maka, M. Makan, E. K. Makimbetov, C. Manda, N. M. Begue, L. Mason, J. O. Mason III, I. O. Matende, M. Materin, C. C. D. S. Mattosinho, M. Matua, I. Mayet, F. B. Mbumba, J. D. McKenzie, A. Medina-Sanson, A. Mehrvar, A. A. Mengesha, V. Menon, G. J. V. D. Mercado, M. B. Mets, E. Midena, D. K. C. Mishra, F. G. Mndeme, A. A. Mohamedani, M. T. Mohammad, A. C. Moll, M. M. Montero, R. A. Morales, C. Moreira, P. Mruthyunjaya, M. S. Msina, G. Msukwa, S. S. Mudaliar, K. I. Muma, F. L. Munier, G. Murgoi, T. G. Murray, K. O. Musa, A. Mushtaq, H. Mustak, O. M. Muyen, G. Naidu, A. G. Nair, L. Naumenko, P. A. N. Roth, Y. M. Nency, V. Neroev, H. Ngo, R. M. Nieves, M. Nikitovic, E. D. Nkanga, H. Nkumbe, M. Nuruddin, M. Nyaywa, G. Obono-Obiang, N. C. Oguego, A. Olechowski, S. C. N. Oliver, P. Osei-Bonsu, D. Ossandon, M. A. Paez-Escamilla, H. Pagarra, S. L. Painter, V. Paintsil, L. Paiva, B. P. Pal, M. S. Palanivelu, R. Papyan, R. Parrozzani, M. Parulekar, C. R. P. Morales, K. E. Paton, K. Pawinska-Wasikowska, J. Pe’er, A. Peña, S. Peric, C. T. M. Pham, R. Philbert, D. A. Plager, P. Pochop, R. A. Polania, V. G. Polyakov, M. T. Pompe, J. J. Pons, D. Prat, V. Prom, I. Purwanto, A. O. Qadir, S. Qayyum, J. Qian, A. Rahman, S. Rahman, J. Rahmat, P. Rajkarnikar, R. Ramanjulu, A. Ramasubramanian, M. A. Ramirez-Ortiz, L. Raobela, R. Rashid, M. A. Reddy, E. Reich, L. A. Renner, D. Reynders, D. Ribadu, M. M. Riheia, P. Ritter-Sovinz, D. Rojanaporn, L. Romero, S. R. Roy, R. H. Saab, S. Saakyan, A. H. Sabhan, M. S. Sagoo, A. M. A. Said, R. Saiju, B. Salas, S. S. R. Pacheco, G. L. Sánchez, P. Sayalith, T. A. Scanlan, A. C. Schefler, J. Schoeman, A. Sedaghat, S. Seregard, R. Seth, A. S. Shah, S. A. Shakoor, M. K. Sharma, S. T. Sherief, N. G. Shetye, C. L. Shields, S. N. Siddiqui, S. S. Cheikh, S. Silva, A. D. Singh, N. Singh, U. Singh, P. Singha, R. S. Sitorus, A. H. Skalet, H. D. Soebagjo, T. Sorochynska, G. Ssali, A. W. Stacey, S. E. Staffieri, E. D. Stahl, C. Stathopoulos, B. S. Kranjc, D. K. Stones, C. Strahlendorf, M. E. C. Suarez, S. Sultana, X. Sun, M. Sundy, R. Superstein, E. Supriyadi, S. Surukrattanaskul, S. Suzuki, K. Svojgr, F. Sylla, G. Tamamyan, D. Tan, A. Tandili, F. F. T. Leiva, M. Tashvighi, B. Tateshi, E. S. Tehuteru, L. F. Teixeira, K. H. Teh, T. Theophile, H. Toledano, D. L. Trang, F. Traoré, S. Trichaiyaporn, S. Tuncer, H. Tyau-Tyau, A. B. Umar, E. Unal, O. E. Uner, S. F. Urbak, T. L. Ushakova, R. H. Usmanov, S. Valeina, M. van Hoefen Wijsard, A. Varadisai, L. Vasquez, L. O. Vaughan, N. V. Veleva-Krasteva, N. Verma, A. A. Victor, M. Viksnins, E. G. V. Chafla, V. Vishnevskia-Dai, T. Vora, A. E. Wachtel, W. Wackernagel, K. Waddell, P. D. Wade, A. H. Wali, Y.-Z. Wang, A. Weiss, M. W. Wilson, A. D. C. Wime, A. Wiwatwongwana, D. Wiwatwongwana, C. W. Dod, P. Wongwai, D. Xiang, Y. Xiao, J. C. Yam, H. Yang, J. M. Yanga, M. A. Yaqub, V. A. Yarovaya, A. A. Yarovoy, H. Ye, Y. A. Yousef, P. Yuliawati, A. M. Z. López, E. Zein, C. Zhang, Y. Zhang, J. Zhao, X. Zheng, K. Zhilyaeva, N. Zia, O. A. O. Ziko, M. Zondervan, R. Bowman, Global retinoblastoma presentation and analysis by national income level. JAMA Oncol. 6, 1–12 (2020). 10.1001/jamaoncol.2019.6716 PMC7047856 32105305

[2] H. E. Grossniklaus, Retinoblastoma. Fifty years of progress. The LXXI Edward Jackson memorial lecture. Am. J. Ophthalmol. 158, 875–891.e1 (2014). 25065496 10.1016/j.ajo.2014.07.025 PMC4250440

[3] S. H. Friend, R. Bernards, S. Rogelj, R. A. Weinberg, J. M. Rapaport, D. M. Albert, T. P. Dryja, A human DNA segment with properties of the gene that predisposes to retinoblastoma and osteosarcoma. Nature 323, 643–646 (1986). 2877398 10.1038/323643a0

[4] X. L. Xu, H. P. Singh, L. Wang, D. L. Qi, B. K. Poulos, D. H. Abramson, S. C. Jhanwar, D. Cobrinik, Rb suppresses human cone-precursor-derived retinoblastoma tumours. Nature 514, 385–388 (2014). 25252974 10.1038/nature13813 PMC4232224

[5] T. W. Corson, B. L. Gallie, One hit, two hits, three hits, more? Genomic changes in the development of retinoblastoma. Genes Chromosomes Cancer 46, 617–634 (2007). 17437278 10.1002/gcc.20457

[6] H. Dimaras, V. Khetan, W. Halliday, M. Orlic, N. L. Prigoda, B. Piovesan, P. Marrano, T. W. Corson, R. C. Eagle Jr., J. A. Squire, B. L. Gallie, Loss of RB1 induces non-proliferative retinoma: Increasing genomic instability correlates with progression to retinoblastoma. Hum. Mol. Genet. 17, 1363–1372 (2008). 18211953 10.1093/hmg/ddn024

[7] I. E. Kooi, B. M. Mol, M. P. G. Massink, N. Ameziane, H. Meijers-Heijboer, C. J. Dommering, S. E. van Mil, Y. de Vries, A. H. van der Hout, G. J. L. Kaspers, A. C. Moll, H. te Riele, J. Cloos, J. C. Dorsman, Somatic genomic alterations in retinoblastoma beyond RB1 are rare and limited to copy number changes. Sci. Rep. 6, 25264 (2016). 27126562 10.1038/srep25264 PMC4850475

[8] K. Sampieri, M. Amenduni, F. T. Papa, E. Katzaki, M. A. Mencarelli, A. Marozza, M. C. Epistolato, P. Toti, S. Lazzi, M. Bruttini, R. de Filippis, S. de Francesco, I. Longo, I. Meloni, F. Mari, A. Acquaviva, T. Hadjistilianou, A. Renieri, F. Ariani, Array comparative genomic hybridization in retinoma and retinoblastoma tissues. Cancer Sci. 100, 465–471 (2009). 19183342 10.1111/j.1349-7006.2008.01070.x PMC11159683

[9] N. A. Laurie, S. L. Donovan, C. S. Shih, J. Zhang, N. Mills, C. Fuller, A. Teunisse, S. Lam, Y. Ramos, A. Mohan, D. Johnson, M. Wilson, C. Rodriguez-Galindo, M. Quarto, S. Francoz, S. M. Mendrysa, R. Kiplin Guy, J. C. Marine, A. G. Jochemsen, M. A. Dyer, Inactivation of the p53 pathway in retinoblastoma. Nature 444, 61–66 (2006). 17080083 10.1038/nature05194

[10] J. H. Francis, A. L. Richards, D. L. Mandelker, M. F. Berger, M. F. Walsh, I. J. Dunkel, M. T. A. Donoghue, D. H. Abramson, Molecular changes in retinoblastoma beyond RB1: Findings from next-generation sequencing. Cancers (Basel) 13, 149 (2021). 33466343 10.3390/cancers13010149 PMC7796332

[11] H. R. Davies, K. D. Broad, Z. Onadim, E. A. Price, X. Zou, I. Sheriff, E. K. Karaa, I. Scheimberg, M. A. Reddy, M. S. Sagoo, S. I. Ohnuma, S. Nik-Zainal, Whole-genome sequencing of retinoblastoma reveals the diversity of rearrangements disrupting RB1 and uncovers a treatment-related mutational signature. Cancers (Basel) 13, 754 (2021). 33670346 10.3390/cancers13040754 PMC7918943

[12] A. R. Afshar, M. Pekmezci, M. M. Bloomer, N. J. Cadenas, M. Stevers, A. Banerjee, R. Roy, A. B. Olshen, J. Van Ziffle, C. Onodera, W. P. Devine, J. P. Grenert, B. C. Bastian, D. A. Solomon, B. E. Damato, Next-generation sequencing of retinoblastoma Identifies pathogenic alterations beyond RB1 inactivation that correlate with aggressive histopathologic features. Ophthalmology 127, 804–813 (2020). 32139107 10.1016/j.ophtha.2019.12.005 PMC7246167

[13] A. Kramer, J. Green, J. Pollard Jr., S. Tugendreich, Causal analysis approaches in ingenuity pathway analysis. Bioinformatics 30, 523–530 (2014). 24336805 10.1093/bioinformatics/btt703 PMC3928520

[14] Y. Jiang, F. Qian, X. Bai, Y. Liu, Q. Wang, B. Ai, X. Han, S. Shi, J. Zhang, X. Li, Z. Tang, Q. Pan, Y. Wang, F. Wang, C. Li, SEdb: A comprehensive human super-enhancer database. Nucleic Acids Res. 47, D235–D243 (2019). 30371817 10.1093/nar/gky1025 PMC6323980

[15] B. Xiao, J. Spencer, A. Clements, N. Ali-Khan, S. Mittnacht, C. Broceño, M. Burghammer, A. Perrakis, R. Marmorstein, S. J. Gamblin, Crystal structure of the retinoblastoma tumor suppressor protein bound to E2F and the molecular basis of its regulation. Proc. Natl. Acad. Sci. U.S.A. 100, 2363–2368 (2003). 12598654 10.1073/pnas.0436813100 PMC151346

[16] C. Lee, J. H. Chang, H. S. Lee, Y. Cho, Structural basis for the recognition of the E2F transactivation domain by the retinoblastoma tumor suppressor. Genes Dev. 16, 3199–3212 (2002). 12502741 10.1101/gad.1046102 PMC187509

[17] W. D. Cress, D. G. Johnson, J. R. Nevins, A genetic analysis of the E2F1 gene distinguishes regulation by Rb, p107, and adenovirus E4. Mol. Cell. Biol. 13, 6314–6325 (1993). 8413230 10.1128/mcb.13.10.6314-6325.1993 PMC364690

[18] H. Dinkel, K. van Roey, S. Michael, M. Kumar, B. Uyar, B. Altenberg, V. Milchevskaya, M. Schneider, H. Kühn, A. Behrendt, S. L. Dahl, V. Damerell, S. Diebel, S. Kalman, S. Klein, A. C. Knudsen, C. Mäder, S. Merrill, A. Staudt, V. Thiel, L. Welti, N. E. Davey, F. Diella, T. J. Gibson, ELM 2016—Data update and new functionality of the eukaryotic linear motif resource. Nucleic Acids Res. 44, D294–D300 (2016). 26615199 10.1093/nar/gkv1291 PMC4702912

[19] R. Sladek, J. A. Bader, V. Giguere, The orphan nuclear receptor estrogen-related receptor alpha is a transcriptional regulator of the human medium-chain acyl coenzyme A dehydrogenase gene. Mol. Cell. Biol. 17, 5400–5409 (1997). 9271417 10.1128/mcb.17.9.5400 PMC232390

[20] E. Audet-Walsh, T. Yee, S. McGuirk, M. Vernier, C. Ouellet, J. St-Pierre, V. Giguère, Androgen-dependent repression of ERRγ reprograms metabolism in prostate cancer. Cancer Res. 77, 378–389 (2017). 27821488 10.1158/0008-5472.CAN-16-1204

[21] T. Sakamoto, T. R. Matsuura, S. Wan, D. M. Ryba, J. Kim, K. J. Won, L. Lai, C. Petucci, N. Petrenko, K. Musunuru, R. B. Vega, D. P. Kelly, A critical role for estrogen-related receptor signaling in cardiac maturation. Circ. Res. 126, 1685–1702 (2020). 10.1161/CIRCRESAHA.119.316100 PMC7274895 32212902

[22] S. Yu, X. Wang, C. F. Ng, S. Chen, F. L. Chan, ERRgamma suppresses cell proliferation and tumor growth of androgen-sensitive and androgen-insensitive prostate cancer cells and its implication as a therapeutic target for prostate cancer. Cancer Res. 67, 4904–4914 (2007). 17510420 10.1158/0008-5472.CAN-06-3855

[23] J. Y. Do, Y. K. Choi, H. Kook, K. Suk, I. K. Lee, D. H. Park, Retinal hypoxia induces vascular endothelial growth factor through induction of estrogen-related receptor γ. Biochem. Biophys. Res. Commun. 460, 457–463 (2015). 25796334 10.1016/j.bbrc.2015.03.055

[24] P. Kumar, C. R. Mendelson, Estrogen-related receptor gamma (ERRgamma) mediates oxygen-dependent induction of aromatase (CYP19) gene expression during human trophoblast differentiation. Mol. Endocrinol. 25, 1513–1526 (2011). 21757507 10.1210/me.2011-1012 PMC3165917

[25] E. Y. H. Chao, J. L. Collins, S. Gaillard, A. B. Miller, L. Wang, L. A. Orband-Miller, R. T. Nolte, D. P. McDonnell, T. M. Willson, W. J. Zuercher, Structure-guided synthesis of tamoxifen analogs with improved selectivity for the orphan ERRgamma. Bioorg. Med. Chem. Lett. 16, 821–824 (2006). 16307879 10.1016/j.bmcl.2005.11.030

[26] P. T. Finger, J. W. Harbour, Z. A. Karcioglu, Risk factors for metastasis in retinoblastoma. Surv. Ophthalmol. 47, 1–16 (2002). 11801265 10.1016/s0039-6257(01)00279-x

[27] J. A. Simon, R. E. Kingston, Mechanisms of polycomb gene silencing: Knowns and unknowns. Nat. Rev. Mol. Cell Biol. 10, 697–708 (2009). 19738629 10.1038/nrm2763

[28] D. Ng, N. Thakker, C. M. Corcoran, D. Donnai, R. Perveen, A. Schneider, D. W. Hadley, C. Tifft, L. Zhang, A. O. M. Wilkie, J. J. van der Smagt, R. J. Gorlin, S. M. Burgess, V. J. Bardwell, G. C. M. Black, L. G. Biesecker, Oculofaciocardiodental and Lenz microphthalmia syndromes result from distinct classes of mutations in BCOR. Nat. Genet. 36, 411–416 (2004). 15004558 10.1038/ng1321

[29] Y. Yamamoto, A. Abe, N. Emi, Clarifying the impact of polycomb complex component disruption in human cancers. Mol. Cancer Res. 12, 479–484 (2014). 24515802 10.1158/1541-7786.MCR-13-0596

[30] I. E. Kooi, B. M. Mol, M. P. G. Massink, M. C. de Jong, P. de Graaf, P. van der Valk, H. Meijers-Heijboer, G. J. L. Kaspers, A. C. Moll, H. te Riele, J. Cloos, J. C. Dorsman, A meta-analysis of retinoblastoma copy numbers refines the list of possible driver genes involved in tumor progression. PLOS ONE 11, e0153323 (2016). 27115612 10.1371/journal.pone.0153323 PMC4846005

[31] I. Hermans-Borgmeyer, U. Susens, U. Borgmeyer, Developmental expression of the estrogen receptor-related receptor gamma in the nervous system during mouse embryogenesis. Mech. Dev. 97, 197–199 (2000). 11025225 10.1016/s0925-4773(00)00422-6

[32] L. Pei, Y. Mu, M. Leblanc, W. Alaynick, G. D. Barish, M. Pankratz, T. W. Tseng, S. Kaufman, C. Liddle, R. T. Yu, M. Downes, S. L. Pfaff, J. Auwerx, F. H. Gage, R. M. Evans, Dependence of hippocampal function on ERRγ-regulated mitochondrial metabolism. Cell Metab. 21, 628–636 (2015). 25863252 10.1016/j.cmet.2015.03.004 PMC4393848

[33] A. Ao, H. Wang, S. Kamarajugadda, J. Lu, Involvement of estrogen-related receptors in transcriptional response to hypoxia and growth of solid tumors. Proc. Natl. Acad. Sci. U.S.A. 105, 7821–7826 (2008). 18509053 10.1073/pnas.0711677105 PMC2409389

[34] P. A. Ruzycki, X. Zhang, S. Chen, CRX directs photoreceptor differentiation by accelerating chromatin remodeling at specific target sites. Epigenetics Chromatin 11, 42 (2018). 30068366 10.1186/s13072-018-0212-2 PMC6069558

[35] T. J. Cherry, S. Wang, I. Bormuth, M. Schwab, J. Olson, C. L. Cepko, NeuroD factors regulate cell fate and neurite stratification in the developing retina. J. Neurosci. 31, 7365–7379 (2011). 21593321 10.1523/JNEUROSCI.2555-10.2011 PMC3135085

[36] R. Balasubramanian, A. Bui, Q. Ding, L. Gan, Expression of LIM-homeodomain transcription factors in the developing and mature mouse retina. Gene Expr. Patterns 14, 1–8 (2014). 24333658 10.1016/j.gep.2013.12.001 PMC3921069

[37] H. T. Kim, S. J. Kim, Y. I. Sohn, S. S. Paik, R. Caplette, M. Simonutti, K. H. Moon, E. J. Lee, K. W. Min, M. J. Kim, D. G. Lee, A. Simeone, T. Lamonerie, T. Furukawa, J. S. Choi, H. S. Kweon, S. Picaud, I. B. Kim, M. Shong, J. W. Kim, Mitochondrial protection by exogenous Otx2 in mouse retinal neurons. Cell Rep. 13, 990–1002 (2015). 26565912 10.1016/j.celrep.2015.09.075

[38] H. Yamamoto, T. Kon, Y. Omori, T. Furukawa, Functional and evolutionary diversification of Otx2 and Crx in vertebrate retinal photoreceptor and bipolar cell development. Cell Rep. 30, 658–671.e5 (2020). 31968244 10.1016/j.celrep.2019.12.072

[39] Y. Satou, K. Minami, E. Hosono, H. Okada, Y. Yasuoka, T. Shibano, T. Tanaka, M. Taira, Phosphorylation states change Otx2 activity for cell proliferation and patterning in the Xenopus embryo. Development 145, dev159640 (2018). 29440302 10.1242/dev.159640

[40] J. Li, C. Di, J. Jing, Q. Di, J. Nakhla, D. C. Adamson, OTX2 is a therapeutic target for retinoblastoma and may function as a common factor between C-MYC, CRX, and phosphorylated RB pathways. Int. J. Oncol. 47, 1703–1710 (2015). 26397460 10.3892/ijo.2015.3179

[41] D. D. Glubrecht, J. H. Kim, L. Russell, J. S. Bamforth, R. Godbout, Differential CRX and OTX2 expression in human retina and retinoblastoma. J. Neurochem. 111, 250–263 (2009). 19686387 10.1111/j.1471-4159.2009.06322.x PMC3726384

[42] J. S. Joyal, M. L. Gantner, L. E. H. Smith, Retinal energy demands control vascular supply of the retina in development and disease: The role of neuronal lipid and glucose metabolism. Prog. Retin. Eye Res. 64, 131–156 (2018). 29175509 10.1016/j.preteyeres.2017.11.002 PMC5963988

[43] N. D. Wangsa-Wirawan, R. A. Linsenmeier, Retinal oxygen: Fundamental and clinical aspects. Arch. Ophthalmol. 121, 547–557 (2003). 12695252 10.1001/archopht.121.4.547

[44] Q. Yang, A. Tripathy, W. Yu, C. G. Eberhart, L. Asnaghi, Hypoxia inhibits growth, proliferation, and increases response to chemotherapy in retinoblastoma cells. Exp. Eye Res. 162, 48–61 (2017). 28689747 10.1016/j.exer.2017.07.001

[45] J. Sudhakar, N. Venkatesan, S. Lakshmanan, V. Khetan, S. Krishnakumar, J. Biswas, Hypoxic tumor microenvironment in advanced retinoblastoma. Pediatr. Blood Cancer 60, 1598–1601 (2013). 23804414 10.1002/pbc.24599

[46] M. N. Burnier, I. W. McLean, L. E. Zimmerman, S. H. Rosenberg, Retinoblastoma. The relationship of proliferating cells to blood vessels. Invest. Ophthalmol. Vis. Sci. 31, 2037–2040 (1990). 2211000

[47] U. Winter, R. Aschero, F. Fuentes, F. Buontempo, S. Zugbi, M. Sgroi, C. Sampor, D. Abramson, A. Carcaboso, P. Schaiquevich, Tridimensional retinoblastoma cultures as vitreous seeds models for live-cell imaging of chemotherapy penetration. Int. J. Mol. Sci. 20, 1077 (2019). 30832308 10.3390/ijms20051077 PMC6429414

[48] K. Gündüz, I. Günalp, N. Yalçindağ, E. Unal, N. Taçyildiz, E. Erden, P. O. Geyik, Causes of chemoreduction failure in retinoblastoma and analysis of associated factors leading to eventual treatment with external beam radiotherapy and enucleation. Ophthalmology 111, 1917–1924 (2004). 15465557 10.1016/j.ophtha.2004.04.016

[49] B. F. Fernandes, J. Coates, A. N. Odashiro, C. Quezada, A. Huynh, P. R. Odashiro, M. Odashiro, M. N. Burnier Jr., Hypoxia-inducible factor-1α and its role in the proliferation of retinoblastoma cells. Pathol. Oncol. Res. 20, 557–563 (2014). 24338218 10.1007/s12253-013-9728-8

[50] Y. Y. Li, Y. L. Zheng, Hypoxia promotes invasion of retinoblastoma cells in vitro by upregulating HIF-1α/MMP9 signaling pathway. Eur. Rev. Med. Pharmacol. Sci. 21, 5361–5369 (2017). 29243776 10.26355/eurrev_201712_13921

[51] L. E. Mose, M. D. Wilkerson, D. N. Hayes, C. M. Perou, J. S. Parker, ABRA: Improved coding indel detection via assembly-based realignment. Bioinformatics 30, 2813–2815 (2014). 24907369 10.1093/bioinformatics/btu376 PMC4173014

[52] K. Cibulskis, M. S. Lawrence, S. L. Carter, A. Sivachenko, D. Jaffe, C. Sougnez, S. Gabriel, M. Meyerson, E. S. Lander, G. Getz, Sensitive detection of somatic point mutations in impure and heterogeneous cancer samples. Nat. Biotechnol. 31, 213–219 (2013). 23396013 10.1038/nbt.2514 PMC3833702

[53] K. Wang, M. Li, H. Hakonarson, ANNOVAR: Functional annotation of genetic variants from high-throughput sequencing data. Nucleic Acids Res. 38, e164 (2010). 20601685 10.1093/nar/gkq603 PMC2938201

[54] I. A. Adzhubei, S. Schmidt, L. Peshkin, V. E. Ramensky, A. Gerasimova, P. Bork, A. S. Kondrashov, S. R. Sunyaev, A method and server for predicting damaging missense mutations. Nat. Methods 7, 248–249 (2010). 20354512 10.1038/nmeth0410-248 PMC2855889

[55] H. A. Shihab, J. Gough, D. N. Cooper, I. N. Day, T. R. Gaunt, Predicting the functional consequences of cancer-associated amino acid substitutions. Bioinformatics 29, 1504–1510 (2013). 23620363 10.1093/bioinformatics/btt182 PMC3673218

[56] C. Dong, P. Wei, X. Jian, R. Gibbs, E. Boerwinkle, K. Wang, X. Liu, Comparison and integration of deleteriousness prediction methods for nonsynonymous SNVs in whole exome sequencing studies. Hum. Mol. Genet. 24, 2125–2137 (2015). 25552646 10.1093/hmg/ddu733 PMC4375422

[57] C. Shyr, M. Tarailo-Graovac, M. Gottlieb, J. J. Y. Lee, C. van Karnebeek, W. W. Wasserman, FLAGS, frequently mutated genes in public exomes. BMC Med. Genomics 7, 64 (2014). 25466818 10.1186/s12920-014-0064-y PMC4267152

[58] M. S. Lawrence, P. Stojanov, P. Polak, G. V. Kryukov, K. Cibulskis, A. Sivachenko, S. L. Carter, C. Stewart, C. H. Mermel, S. A. Roberts, A. Kiezun, P. S. Hammerman, A. McKenna, Y. Drier, L. Zou, A. H. Ramos, T. J. Pugh, N. Stransky, E. Helman, J. Kim, C. Sougnez, L. Ambrogio, E. Nickerson, E. Shefler, M. L. Cortés, D. Auclair, G. Saksena, D. Voet, M. Noble, D. DiCara, P. Lin, L. Lichtenstein, D. I. Heiman, T. Fennell, M. Imielinski, B. Hernandez, E. Hodis, S. Baca, A. M. Dulak, J. Lohr, D. A. Landau, C. J. Wu, J. Melendez-Zajgla, A. Hidalgo-Miranda, A. Koren, S. A. McCarroll, J. Mora, R. S. Lee, B. Crompton, R. Onofrio, M. Parkin, W. Winckler, K. Ardlie, S. B. Gabriel, C. W. M. Roberts, J. A. Biegel, K. Stegmaier, A. J. Bass, L. A. Garraway, M. Meyerson, T. R. Golub, D. A. Gordenin, S. Sunyaev, E. S. Lander, G. Getz, Mutational heterogeneity in cancer and the search for new cancer-associated genes. Nature 499, 214–218 (2013). 23770567 10.1038/nature12213 PMC3919509

[59] J. Ou, L. J. Zhu, trackViewer: A Bioconductor package for interactive and integrative visualization of multi-omics data. Nat. Methods 16, 453–454 (2019). 31133757 10.1038/s41592-019-0430-y

[60] E. Talevich, A. H. Shain, T. Botton, B. C. Bastian, CNVkit: Genome-wide copy number detection and visualization from targeted DNA sequencing. PLoS Comput. Biol. 12, e1004873 (2016). 27100738 10.1371/journal.pcbi.1004873 PMC4839673

[61] S. Nik-Zainal, P. van Loo, D. C. Wedge, L. B. Alexandrov, C. D. Greenman, K. W. Lau, K. Raine, D. Jones, J. Marshall, M. Ramakrishna, A. Shlien, S. L. Cooke, J. Hinton, A. Menzies, L. A. Stebbings, C. Leroy, M. Jia, R. Rance, L. J. Mudie, S. J. Gamble, P. J. Stephens, S. McLaren, P. S. Tarpey, E. Papaemmanuil, H. R. Davies, I. Varela, D. McBride, G. R. Bignell, K. Leung, A. P. Butler, J. W. Teague, S. Martin, G. Jönsson, O. Mariani, S. Boyault, P. Miron, A. Fatima, A. Langerød, S. A. Aparicio, A. Tutt, A. M. Sieuwerts, Å. Borg, G. Thomas, A. V. Salomon, A. L. Richardson, A. L. Børresen-Dale, P. A. Futreal, M. R. Stratton, P. J. Campbell; Breast Cancer Working Group of the International Cancer Genome Consortium, The life history of 21 breast cancers. Cell 149, 994–1007 (2012). 22608083 10.1016/j.cell.2012.04.023 PMC3428864

[62] C. H. Mermel, S. E. Schumacher, B. Hill, M. L. Meyerson, R. Beroukhim, G. Getz, GISTIC2.0 facilitates sensitive and confident localization of the targets of focal somatic copy-number alteration in human cancers. Genome Biol. 12, R41 (2011). 21527027 10.1186/gb-2011-12-4-r41 PMC3218867

[63] T. Rausch, T. Zichner, A. Schlattl, A. M. Stütz, V. Benes, J. O. Korbel, DELLY: Structural variant discovery by integrated paired-end and split-read analysis. Bioinformatics 28, i333–i339 (2012). 22962449 10.1093/bioinformatics/bts378 PMC3436805

[64] L. A. Seifried, S. Talluri, M. Cecchini, L. M. Julian, J. S. Mymryk, F. A. Dick, pRB-E2F1 complexes are resistant to adenovirus E1A-mediated disruption. J. Virol. 82, 4511–4520 (2008). 18305049 10.1128/JVI.02713-07 PMC2293062

[65] B. J. Girard, T. M. Regan Anderson, S. L. Welch, J. Nicely, V. L. Seewaldt, J. H. Ostrander, Cytoplasmic PELP1 and ERRgamma protect human mammary epithelial cells from Tam-induced cell death. PLOS ONE 10, e0121206 (2015). 25789479 10.1371/journal.pone.0121206 PMC4366195

[66] W. A. Braden, A. K. McClendon, E. S. Knudsen, Cyclin-dependent kinase 4/6 activity is a critical determinant of pre-replication complex assembly. Oncogene 27, 7083–7093 (2008). 18776921 10.1038/onc.2008.319

[67] J. F. Conklin, J. Baker, J. Sage, The RB family is required for the self-renewal and survival of human embryonic stem cells. Nat. Commun. 3, 1244 (2012). 23212373 10.1038/ncomms2254

[68] L. Micallef, P. Rodgers, eulerAPE: Drawing area-proportional 3-venn diagrams using ellipses. PLOS ONE 9, e101717 (2014). 25032825 10.1371/journal.pone.0101717 PMC4102485

[69] S. Kurtenbach, J. W. Harbour, SparK: A publication-quality NGS visualization tool. bioRxiv 845529 (2019).

[70] G. Yu, L. G. Wang, Q. Y. He, ChIPseeker: An R/Bioconductor package for ChIP peak annotation, comparison and visualization. Bioinformatics 31, 2382–2383 (2015). 25765347 10.1093/bioinformatics/btv145

[71] J. R. Conway, A. Lex, N. Gehlenborg, UpSetR: An R package for the visualization of intersecting sets and their properties. Bioinformatics 33, 2938–2940 (2017). 28645171 10.1093/bioinformatics/btx364 PMC5870712

[72] Z. Gu, L. Gu, R. Eils, M. Schlesner, B. Brors, circlize Implements and enhances circular visualization in R. Bioinformatics 30, 2811–2812 (2014). 24930139 10.1093/bioinformatics/btu393

[73] T. Stuart, A. Butler, P. Hoffman, C. Hafemeister, E. Papalexi, W. M. Mauck III, M. Stoeckius, P. Smibert, R. Satija, Comprehensive integration of single cell data. bioRxiv 460147 (2018). 10.1016/j.cell.2019.05.031 PMC6687398 31178118

[74] A. Butler, P. Hoffman, P. Smibert, E. Papalexi, R. Satija, Integrating single-cell transcriptomic data across different conditions, technologies, and species. Nat. Biotechnol. 36, 411–420 (2018). 29608179 10.1038/nbt.4096 PMC6700744

[75] C. S. McGinnis, L. M. Murrow, Z. J. Gartner, DoubletFinder: Doublet detection in single-cell RNA sequencing data using artificial nearest neighbors. Cell Syst. 8, 329–337.e4 (2019). 30954475 10.1016/j.cels.2019.03.003 PMC6853612

[76] E. Becht, L. M. Innes, J. Healy, C.-A. Dutertre, I. W. H. Kwok, L. G. Ng, F. Ginhoux, E. W. Newell, Dimensionality reduction for visualizing single-cell data using UMAP. Nat. Biotechnol. 37, 38–44 (2018). 10.1038/nbt.4314 30531897

[77] V. D. Blondel, J.-L. Guillaume, R. Lambiotte, E. Lefebvre, Fast unfolding of communities in large networks. J. Stat. Mech. Theory Exp. 2008, P10008 (2008).

