# Supplementary material for: RB1 loss triggers dependence on ESRRG in retinoblastoma
Source: Sci Adv. 2022 Aug 19;8(33):eabm8466. doi: 10.1126/sciadv.abm8466 (PMC9390996; doi:10.1126/sciadv.abm8466)
Supplement: Supplementary file 1 — Figs. S1 to S11 [file sciadv.abm8466_sm.pdf]

Science Advances
AAAS

Supplementary Materials for

RB1 loss triggers dependence on ESRRG in retinoblastoma

Matthew G. Field *et al.*

Corresponding author: J. William Harbour, william.harbour@utsouthwestern.edu

Sci. Adv. **8**, eabm8466 (2022)
DOI: 10.1126/sciadv.abm8466

The PDF file includes:

Figs. S1 to S11
Legends for data S1 to S4

Other Supplementary Material for this manuscript includes the following:

Data S1 to S4

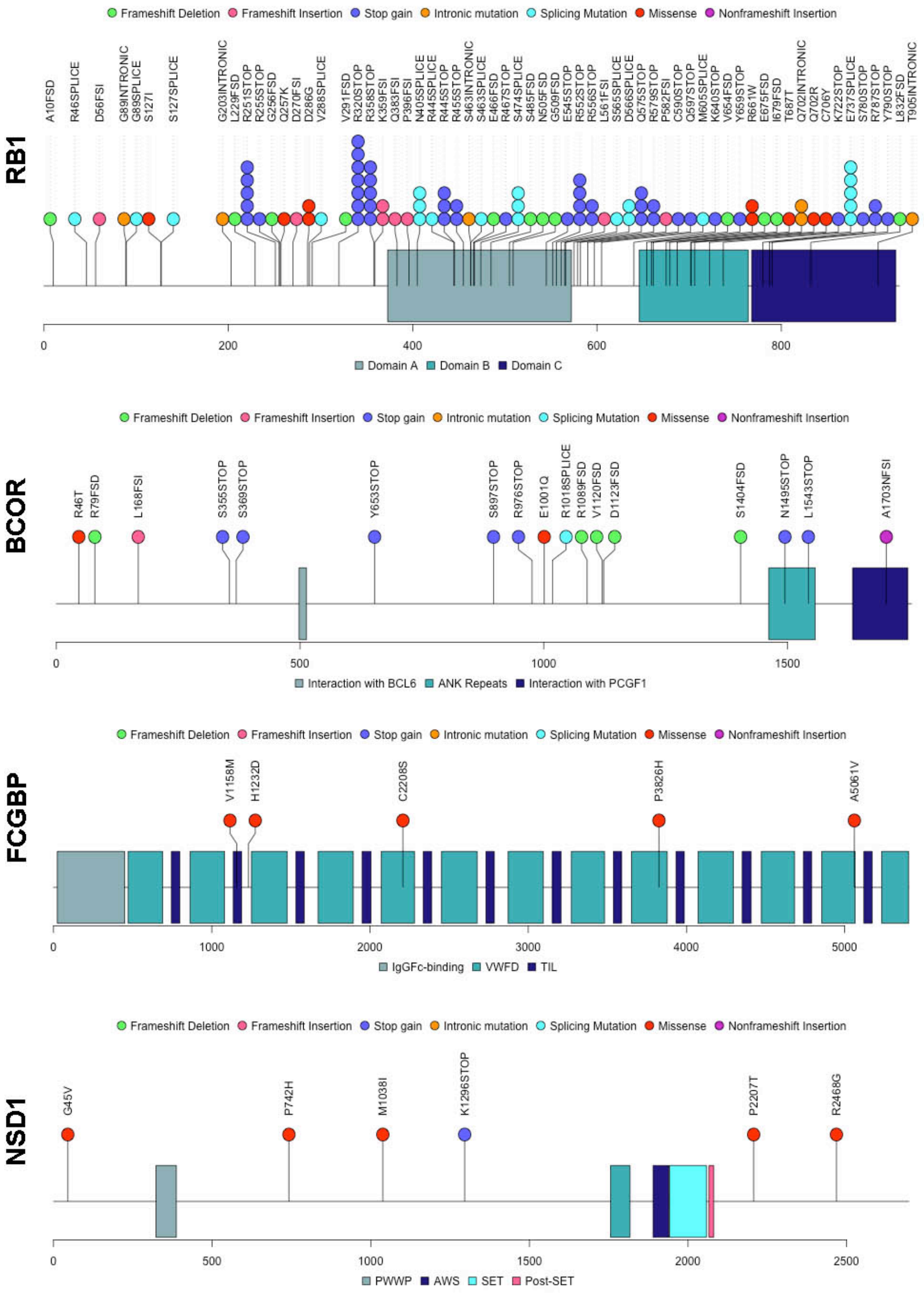

Fig. S1. Lollipop plots of most recurrent gene mutations in 103 whole exome sequencing retinoblastoma samples. Mutations identified in *RB1*, *BCOR*, *FCGBP*, and *NSD1* are plotted along the protein domains and labeled by mutation type.

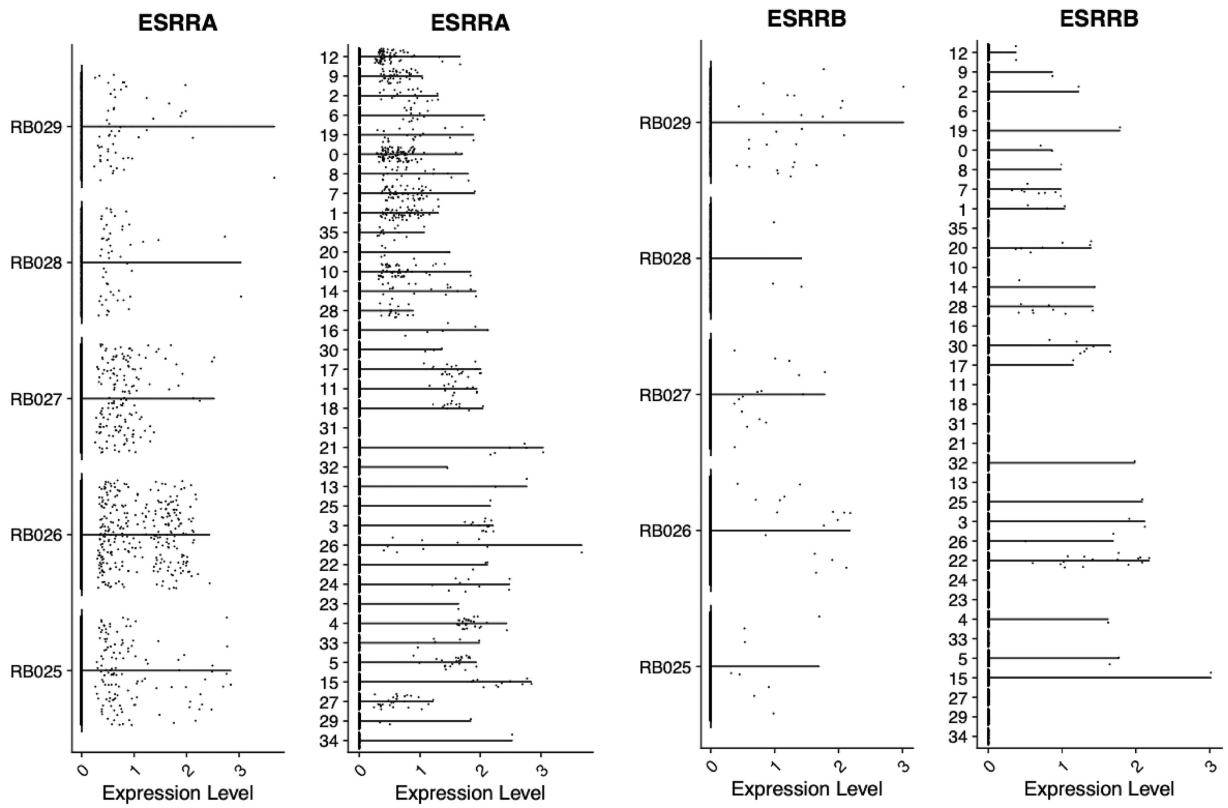

Fig. S2. Single-cell analysis of ESRRA and ESRRB. Violin plots of single-cell RNA sequencing (scRNA-seq) data showing expression of *ESRRA* (**A**) and *ESRRB* (**B**) by tumor sample (left) and Seurat cluster (right).

A

Sample	1q gain	*ESRRG* locus	*ESRRG* mRNA Expression
SJRB001	No	Normal	15.7
SJRB002	No	Normal	41.0
SJRB003	No	INVERSION chr1:180949143 to chr1:233051411	85.8
SJRB004	Yes	TRANSLOCATION chr1:217532626 to chr11:25018985	166.1

B

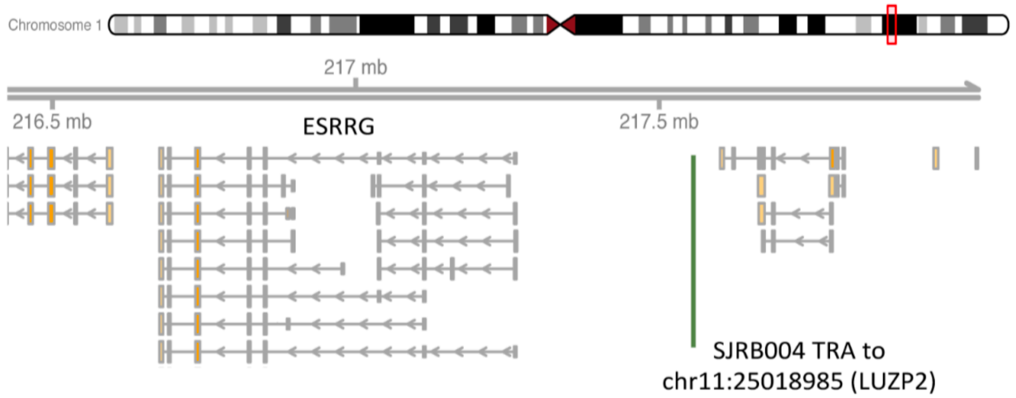

C

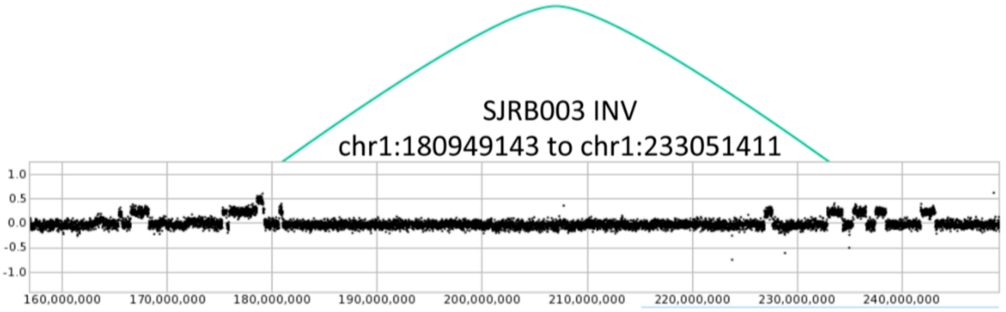

Fig. S3. Identification of complex genomic alterations involving the *ESRRG* locus in whole genome sequencing data from primary retinoblastomas. (**A**) Table summarizing complex rearrangements near the *ESRRG* locus, 1q gain status and *ESRRG* mRNA expression in four Rb samples that underwent whole genome and RNA-sequencing. (**B**) Translocation of *ESRRG* (chr1:217532626) to the *LUZP2* locus on chromosome 11 (chr11:25018985) in sample SJRB004. (**C**) Chromosome 1q inversion (chr1:180949143 to chr1:233051411) involving the *ESRRG* locus in sample SJRB003. CPM, counts per million; INV, inversion; TRA, translocation.

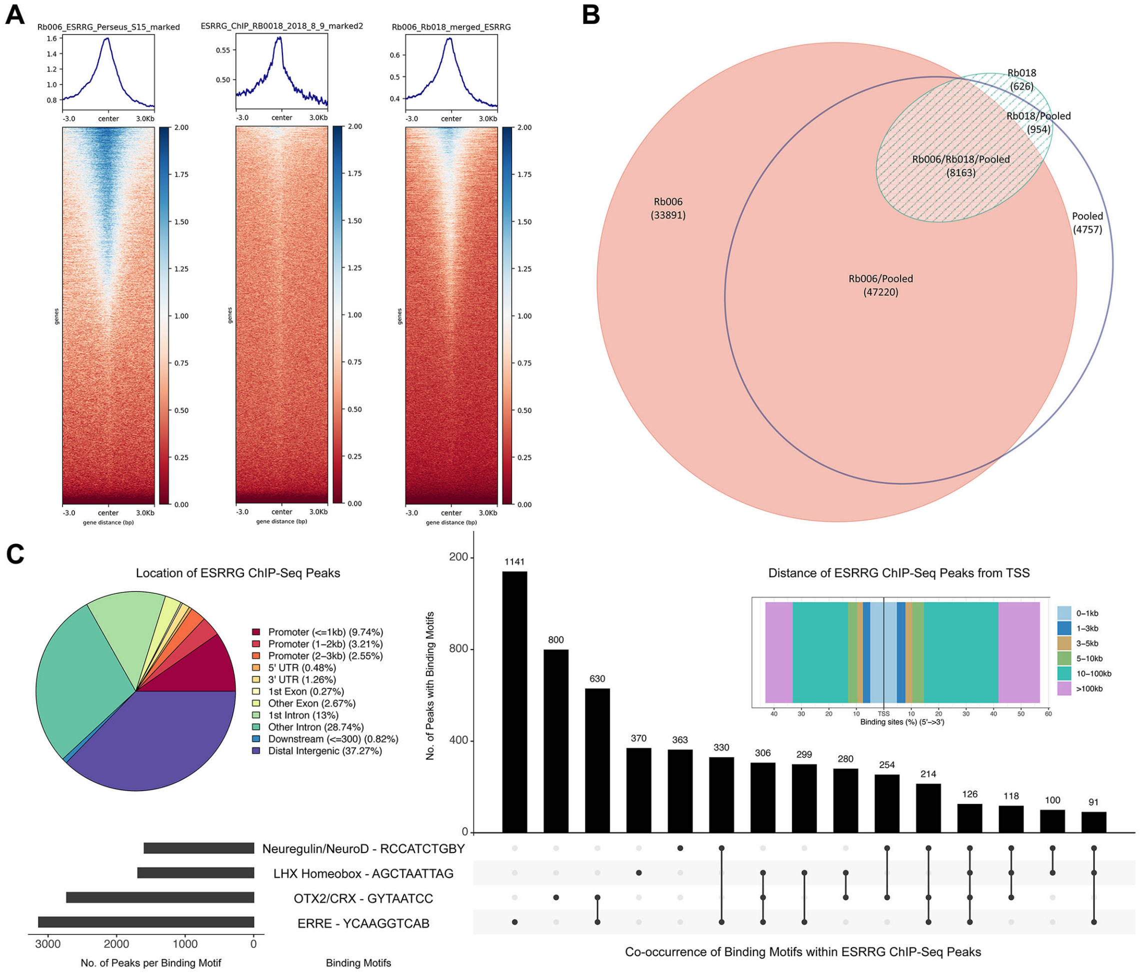

Fig. S4. ESRRG chromatin localization in retinoblastoma cells. (**A**) Heatmaps of called peaks from ChIP-seq pulldown of ESRRG in primary low passage RB006 and RB018 retinoblastoma cells and the two datasets pooled. Peaks ±3kb from transcription start sites (TSS) of coding genes are displayed. (**B**) Euler diagram of overlapping ESRRG ChIP-seq peaks between the 3 datasets. (**C**) Location and motif analysis of the 8163 significantly enriched ($p < 0.001$) ESRRG ChIP-seq peaks shared in common between the 3 datasets. The pie chart displays the percentage of these peaks located within various gene regions. The rectangular plot exhibits the distance of the peaks from the TSS. The bar plot shows the presence and co-occurrence of the most significantly enriched transcription factor binding motifs (FDR < 0.05) found within the ESRRG ChIP-Seq peaks.

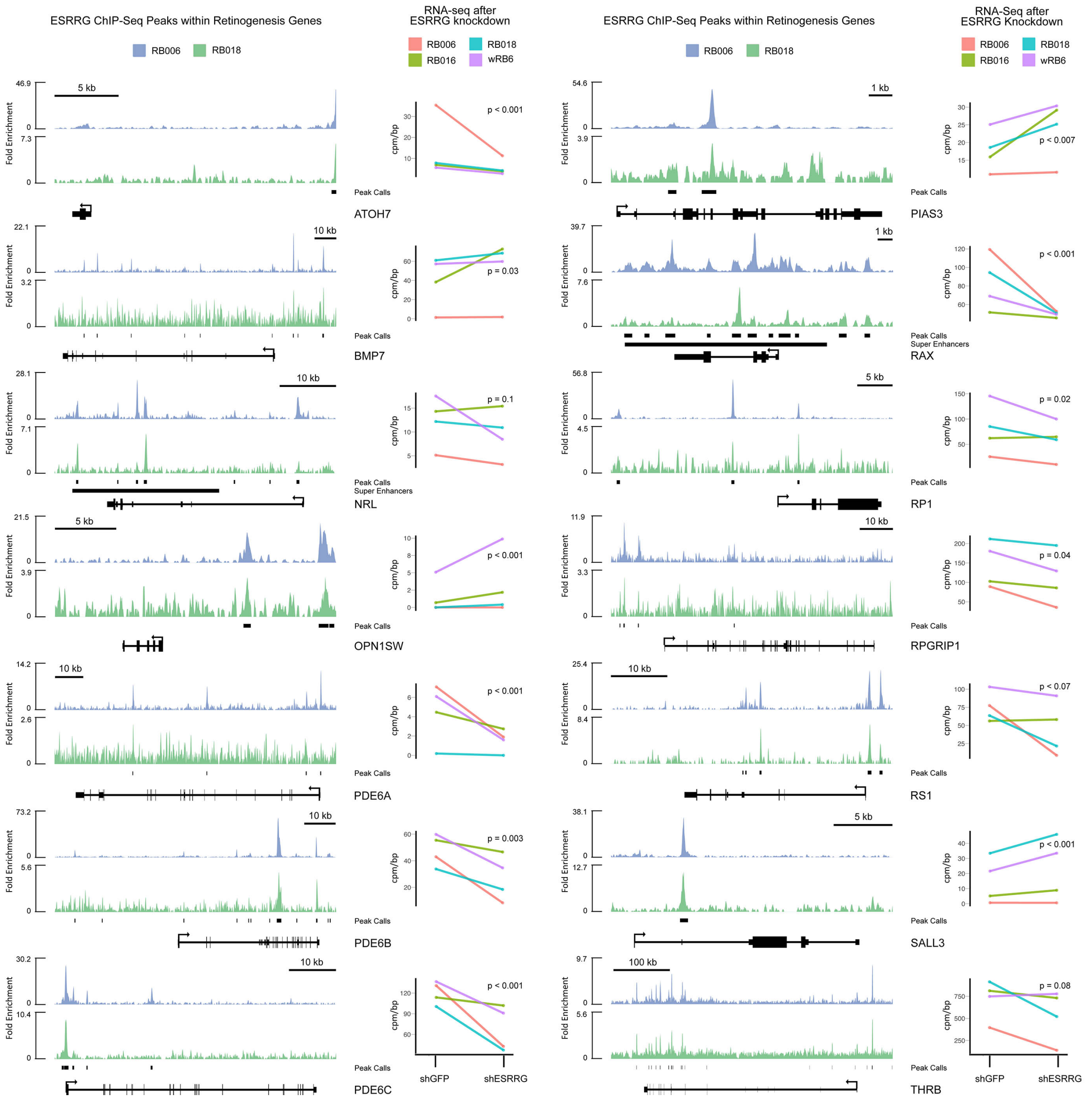

Fig. S5. ESRRG regulates genes involved in retinogenesis. ChIP-seq track plots from ESRRG-pulldown in RB006 (blue) and RB018 (green) retinoblastoma cells are shown for key genes involved in retinal development and differentiation. Peak calls from the pooled dataset, super enhancers, exon and intron locations, and direction of transcription are indicated below the peak plots. Corresponding RNA-seq data for each gene are shown in RB006 (red), RB016 (green), RB018 (blue), and wRB6 (purple) retinoblastoma cells engineered to express shRNA directed against ESRRG (shESRRG) or control (shGFP). P-values were calculated after batch and dispersion correction using EdgeR.

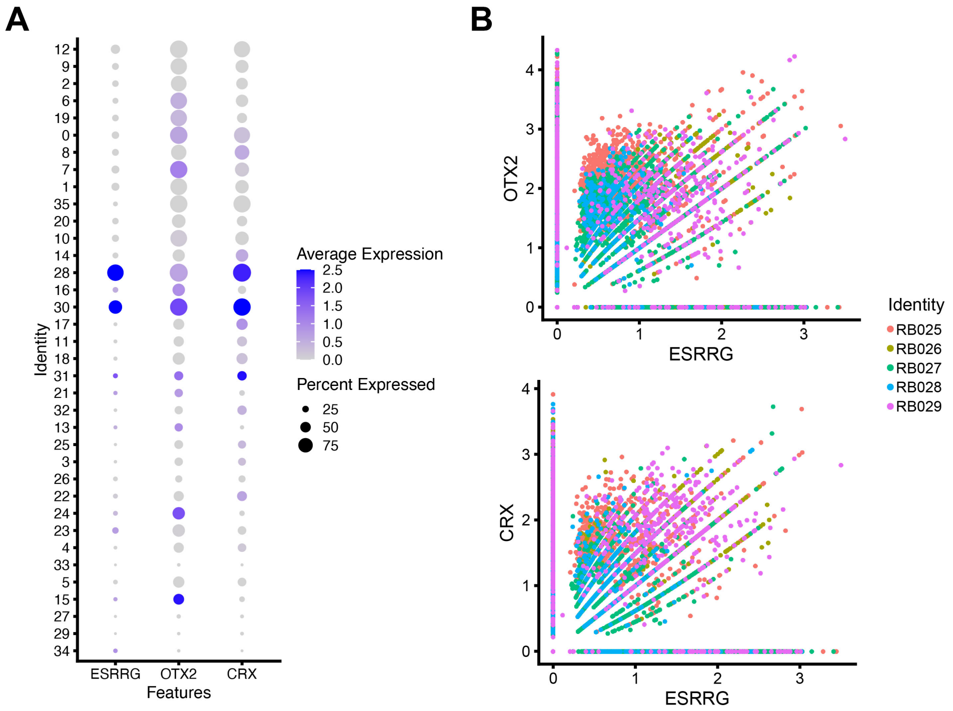

Fig. S6. Correlation of ESRRG expression with CRX and OTX2 in single retinoblastoma cells. (**A**) Dot plot of cell clusters described in Fig. 2C, indicating mRNA expression of *ESRRG*, *CRX* and *OTX2*. (**B**) Scatter plots comparing expression of ESRRG to OTX2 (top) and CRX (bottom).

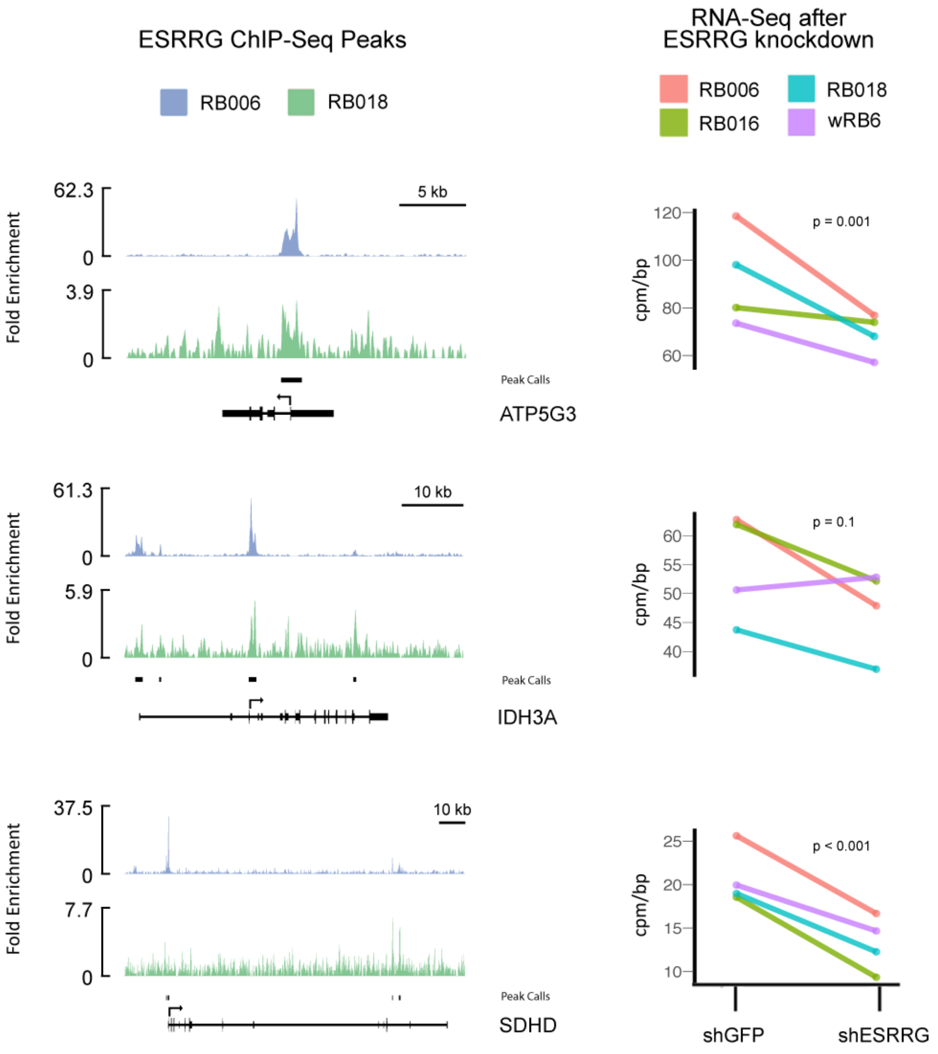

Fig. S7. ESRRG regulates genes involved in oxidative metabolism. ChIP-seq track plots from ESRRG-pulldown in RB006 (blue) and RB018 (green) retinoblastoma cells are shown for key genes involved in energy metabolism (*ATP5G3, IDH3A,* and *SDHD*). Peak calls from the pooled dataset, exon and intron locations, and direction of transcription are indicated below the peak plots. Corresponding RNA-seq data for each gene are shown in RB006 (red), RB016 (green), RB018 (blue), and wRB6 (purple) retinoblastoma cells engineered to express shRNA directed against *ESRRG* (shESRRG) or control (shGFP). P-values were calculated after batch and dispersion correction using EdgeR.

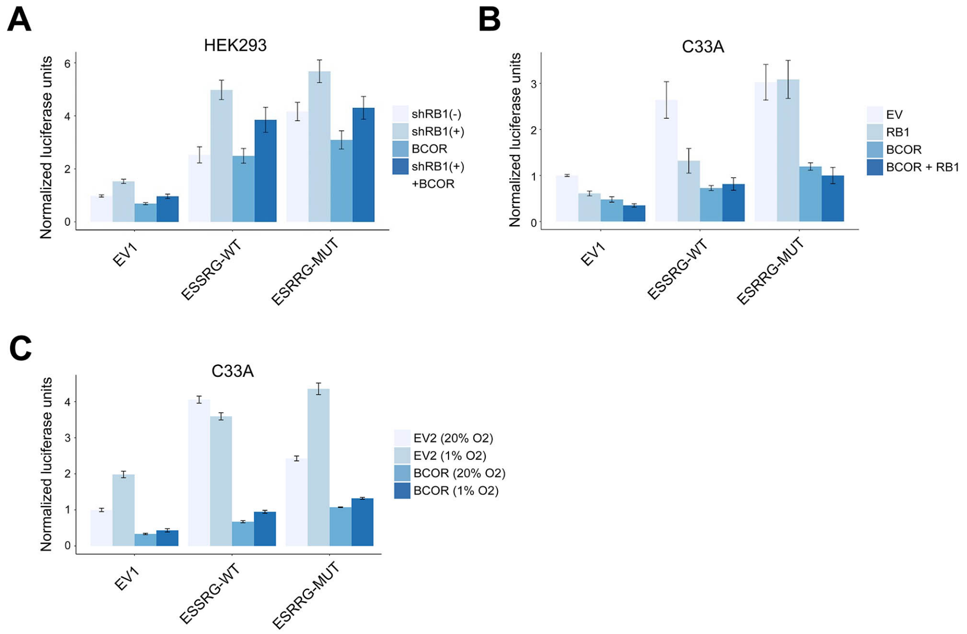

Fig. S8. BCOR represses ERRE promoter independently of RB1. (**A**) Normalized ERRE luciferase reporter activity with or without ectopic expression of V5-ESRRG-WT, V5-ESRRG-MUT, and BCOR, and with or without shRNA-mediated depletion of RB1 (shRB1+ and shRB1-, respectively) in HEK293 cells (n = 12). (**B**) Normalized ERRE luciferase reporter activity with or without ectopic expression of V5-ESRRG-WT, V5-ESRRG-MUT, BCOR, and RB1 in C33A cells (n = 12). (**C**) Normalized ERRE luciferase reporter activity with or without ectopic expression of V5-ESRRG-WT, V5-ESRRG-MUT, and BCOR in C33A cells in hypoxia (1% O2) or normoxia (20% O2)(n = 4). EV, EV1 and EV2, empty vector controls. All data are shown as means ± SEM.

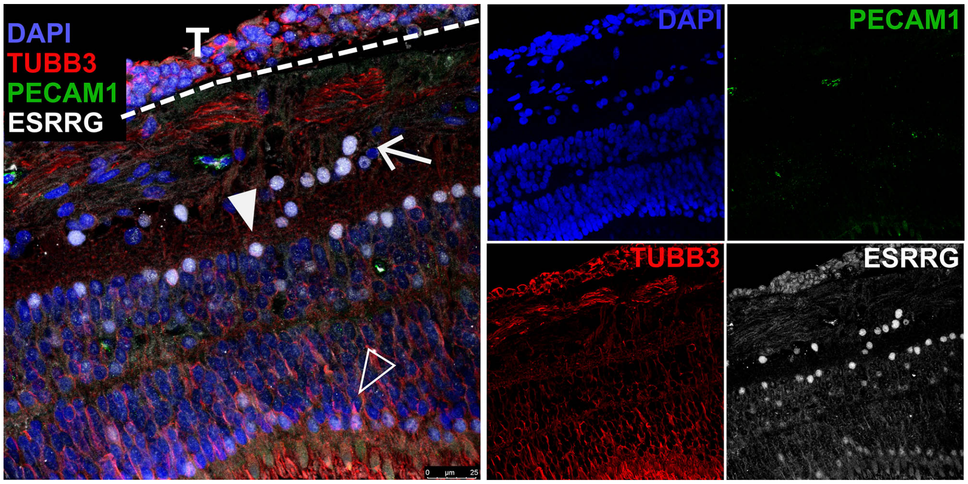

Fig. S9. ESRRG expression in human retina. Multiplexed fluorescence immunohistochemistry of Rb enucleation sample #29-15 in a region of unaffected retina. ESRRG (white), TUBB3 (red), DAPI (blue) and PECAM1 (green). Arrow, retinal ganglion cells; solid arrowhead, amacrine cells; hollow arrowhead, photoreceptors; T, tumor cells located on the inner retinal surface.

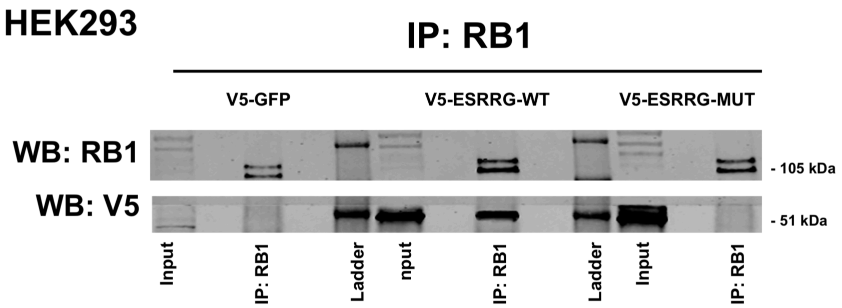

Fig. S10. RB1 interacts with ESRRG. Western blot (WB) for endogenously expressed RB1 and exogenously expressed V5-tagged wildtype ESRRG (V5-ESRRG-WT) or ESRRG with a mutated VXXLYD motif (V5-ESRRG-MUT) in RB1-wildtype HEK293 cells following immunoprecipitation for RB1.

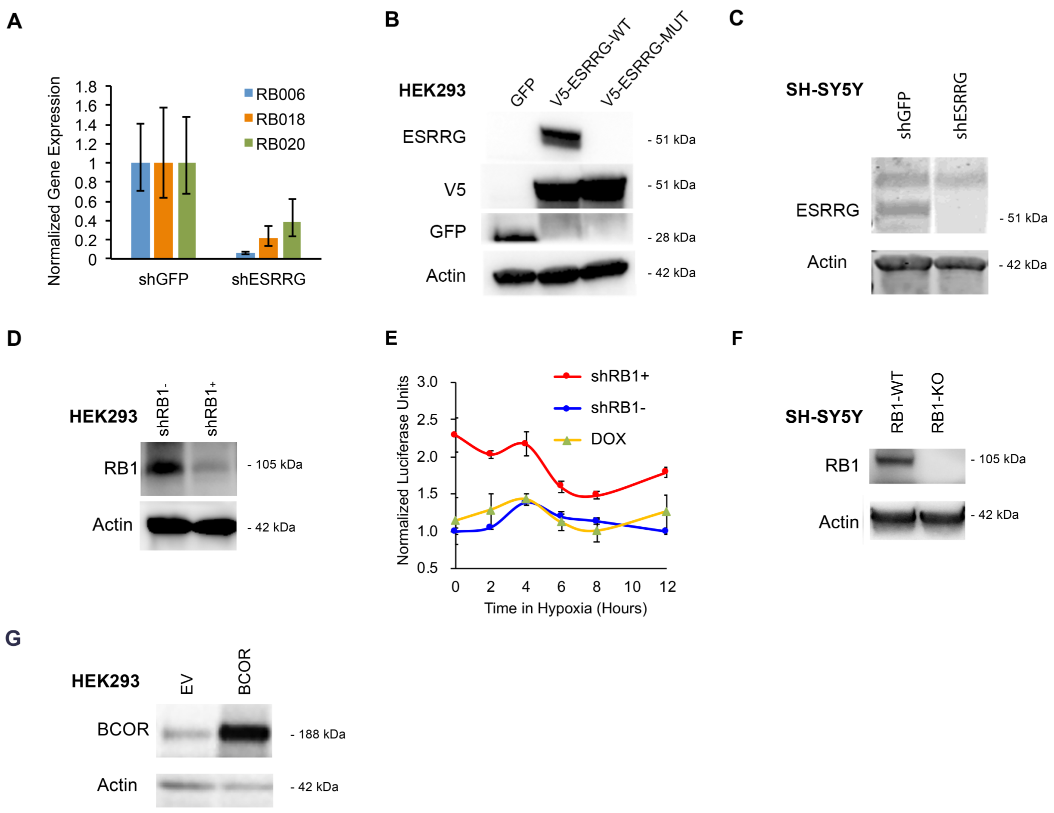

Fig. S11. Control and validation experiments. (**A**) *ESRRG* mRNA expression in RB006, RB018, and RB020 stably expressing shRNA directed against *ESRRG* (shESRRG) or *GFP* control (shGFP) for 7 days (n = 3). The error bars indicate the calculated maximum (RQMax) and minimum (RQMin) expression levels that represent the standard error of the mean expression level (RQ value). (**B**) Western blot (WB) using the R&D (PP- H6812-00) ESRRG antibody could only detect exogenously expressed V5-tagged wildtype ESRRG (V5-ESRRG-WT) and not ESRRG with a mutated VXXLYD motif (V5-ESRRG-MUT), however both constructs were expressed as assessed by V5 antibody immune-detection. V5-tagged GFP protein was used as positive control for V5 antibody. (**C**) WB using the Abcam (ab49129) ESRRG antibody detected endogenously expressed ESRRG. Specificity of the antibody was confirmed by ESRRG knockdown (shESRRG), which abrogated the ESRRG signal. shGFP was used as a negative control. (**D**) WB of HEK293 cells with doxycycline treatment (shRB1+) showed shRNA-mediated knockdown of RB1 compared to un-induced cells (shRB1-) after 24 hours of treatment. (**E**) Normalized ERRE luciferase reporter activity increased with doxycycline-induced knockdown of RB1; HEK293 parental cells treated with doxycycline were used as DOX control and showed no impact of doxycycline on ERRE activity (n = 6). Data are shown as means ± SEM. (**F**) WB for endogenously expressed RB1 in SH-SY5Y neuroblastoma cells with (RB1-KO) or without (RB1-WT) RB1 knockout. (**G**) WB blot of exogenously expressed BCOR compared to empty vector in HEK293 cells.

Data S1. Mutations called in 103 retinoblastoma whole exome sequencing samples.

Data S2. ESRRG ChIP-seq analysis in recently established retinoblastoma cell lines.

Data S3. Integrated analysis of RNA-seq and ChIP-Seq data in recently established retinoblastoma cell lines with or without ESRRG knockdown.

Data S4. List of antibodies and oligonucleotide primer sequences used in this study.
